# Measuring Multi-Joint Stiffness during Single Movements: Numerical Validation of a Novel Time-Frequency Approach

**DOI:** 10.1371/journal.pone.0033086

**Published:** 2012-03-20

**Authors:** Davide Piovesan, Alberto Pierobon, Paul DiZio, James R. Lackner

**Affiliations:** 1 Sensory Motor Performance Program, Rehabilitation Institute of Chicago, Chicago, Illinois, United States of America; 2 Department of Physical Medicine and Rehabilitation, Northwestern University, Chicago, Illinois, United States of America; 3 Ashton Graybiel Spatial Orientation Laboratory, Brandeis University, Waltham, Massachusetts, United States of America; 4 Volen Center for Complex Systems, Brandeis University, Waltham, Massachusetts, United States of America; The University of Western Ontario, Canada

## Abstract

This study presents and validates a Time-Frequency technique for measuring 2-dimensional multijoint arm stiffness throughout a single planar movement as well as during static posture. It is proposed as an alternative to current regressive methods which require numerous repetitions to obtain average stiffness on a small segment of the hand trajectory. The method is based on the analysis of the reassigned spectrogram of the arm's response to impulsive perturbations and can estimate arm stiffness on a trial-by-trial basis. Analytic and empirical methods are first derived and tested through modal analysis on synthetic data. The technique's accuracy and robustness are assessed by modeling the estimation of stiffness time profiles changing at different rates and affected by different noise levels. Our method obtains results comparable with two well-known regressive techniques. We also test how the technique can identify the viscoelastic component of non-linear and higher than second order systems with a non-parametrical approach. The technique proposed here is very impervious to noise and can be used easily for both postural and movement tasks. Estimations of stiffness profiles are possible with only one perturbation, making our method a useful tool for estimating limb stiffness during motor learning and adaptation tasks, and for understanding the modulation of stiffness in individuals with neurodegenerative diseases.

## Introduction

The motor system uses stiffness modulation to maintain stability of the arm during interactions with the environment. It has been experimentally investigated in both postural (i.e. static) and dynamic paradigms. In limb postural experiments, system identification is accomplished using either stochastic perturbations [1], [2], [3], [4], [5] or regressive techniques [6], [7], [8], [9], [10]. Studies that quantify stiffness as a function of hand position along a reaching trajectory typically use regressive procedures [11], [12], [13], [14], [15], [16], [17], [18], [19]. Stochastic methods are based on ensemble techniques [20], [21], [22], [23] and even though they identify the system non-parametrically they require hundreds of perturbed repetitions of the same movement to obtain a reliable estimate of stiffness. These repetitions can induce muscle co-contraction that leads to stiffening of the arm joints [24], and can strongly reduce stretch reflexes [25]. Regressive techniques allow for more natural (not continuously perturbed) movements, but still require many trials to produce reasonable stiffness time-profiles using a parametric approach. A method that could estimate dynamic changes in arm stiffness on a trial-by-trial basis would constitute an ideal tool to monitor changes in stiffness over time.

At present, the majority of regressive techniques to estimate stiffness rely on the calculation of a baseline trajectory followed by the application of a set of mechanical perturbations to the arm. After several repeatable unperturbed trials, a prediction of the unperturbed hand trajectory can be obtained with a time average [14], [15], a look-up table [11] or an auto-regressive (AR) model [17], [18], [19]. Investigators have employed mechanical perturbations of different types, such as force pulses [14], [15], servo-displacements [11], [17], and virtual walls [16], that are generally applied by a robotic manipulandum during randomly selected trials. When a sufficient number of perturbations is delivered in multiple directions at the same point along the arm kinematic profile, stiffness is calculated by means of a regression between the variation of hand kinematics and the forces generated by the perturbation.

Regressive techniques rely on the assumption that unperturbed arm movements are repeatable and that the mechanical characteristics of the arm do not change over a small set of repetitions (ergodicity), To obtain the estimation of the baseline trajectory and a set of perturbation responses with such techniques, a series of measures needs to be taken using the same reproducible kinematic configuration; consequently, the data collection burden can be substantial. If a servo-commanded displacement is used, estimates of stiffness can be done independently of the values of damping and inertia when the perturbation reaches steady state [10], [11], [12], [13], [17], [18], [19], [26]. As a consequence, the required characteristics of the robotic devices can be very demanding. In general, when using displacement perturbations, a very stiff environment must be rendered by the robot to keep the actual displacement of the hand as close as possible to the perturbation imposed and to break the feedback loop between joint torques and joint positions, effectively creating an open-loop system that it is possible to identify [27].

The purpose of the present study is to present a technique for measuring time-varying limb stiffness on a trial-by trial basis. The technique is based on time-frequency domain and modal analysis. It requires neither the assumption of stationarity nor the repeatability of the motor task (ergodicity). To show the utility of the proposed method we compare it with two well known regressive techniques, one using force perturbations [15] and the other displacement perturbations [7], [8], [11]. We demonstrate with synthetic data that our proposed technique produces accurate estimates of time-variant stiffness on a single trial basis, under both static and dynamic conditions.

Time-frequency techniques are relatively new to the field of motor control despite having been widely used in fields such as structural engineering [28], [29], [30], radar, sonar, and medical imaging [31]. They depend on evaluating the location of the maximal energy density of a signal in the time-frequency domain. We applied this approach to measure the response of a mechanical system to a transient perturbation to identify the system features. The versatility of this technique allows for several types of perturbations to be used, including force impulses, hold and release [32], and force steps. Classical regressive methods are limited to estimating an average value of stiffness across several trials; by contrast, our time-frequency technique can estimate the variation of stiffness and damping across trials, thereby providing a tool to study the relationship between stiffness modulation and adaptive learning. The proposed method is non-parametric and we tested it on higher-than-second-order and non-linear systems. Modal analysis was used as a parameter identification method for second-order systems to allow a direct comparison with regressive techniques. Linearity, repeatability of the trajectory, and steady state were not necessary assumptions, and a stiff robot was not required because a free response was measured.

In the following sections, we outline how our method was implemented and tested. First we describe the variational approach we apply to the identification of a non-stationary vibrating mechanical system. Then we explain how the system identification is carried out in the time-frequency analysis by means of a reassigned spectrogram, and how this tool allows a parametric identification of time-varying second order mechanical systems as well as a non-parametric identification of non-linear and higher than second order systems (see “The spectrogram”). We provide a description of the models we used to simulate the behavior of human arm movements, as well as a discussion of the characteristics and limitation of each model (see “Assumptions and possible relaxations”). We then introduce and discuss the assumptions under which our method operates, namely that the system exhibits an oscillatory behavior, the instantaneous resonant frequencies are separable, and the system's stiffness and damping matrices are symmetric, though no assumptions on the relationship between stiffness and damping (e.g. proportional or classical damping) are required.

We then show how the systems' equations are normalized with respect to the inertial matrix (see “Equation normalization”), and how the eigenvectors (see “identification of eigenvectors”) and the stiffness and damping parameters (see “system decoupling and modal analysis”) of a second order, two degree-of-freedom (DOF) system are computed through the implementation of our modal analysis.

We provide all the model parameters used in our simulations (see “Description of the simulation”), including the inertial characteristics, the trajectory followed by the simulated arm, the imposed stiffness and damping profiles that we identified, and the parameters specific to each type of mechanical model. We also provide the characteristics of the perturbations used in our identification method, as well as the parameters used in our implementation of previously proposed regressive techniques, to which our method is compared.

Results of the simulations are then presented. The stiffness and damping parameters identified with our method are shown to be statistically comparable to those identified with regressive techniques. Results of the non-parametrical system identification, that our method allows, are also presented.

## Methods

In this section, we provide a variational description of the mechanical system response that is then used in our time-frequency analysis. When studying the motion of a mechanical system, 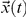 is a vector of generalized position coordinates (angles, Cartesian coordinates, etc.). We can define 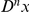 as the set representing the position coordinates and their derivatives with respect to time up to the 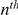 order so that 
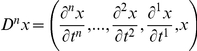
, in general 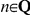 [33].

A mechanical system must comply with the Lagrange–d'Alembert principle so that
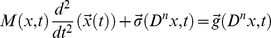
(1)where 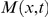 is the inertial matrix of the system in the chosen coordinate frame, 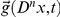 is the external force field, and 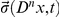 is the internal force field generated by the mechanical network [33]. The goal is to identify the features of the unknown internal force field 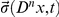.

Since 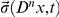 is generally a non-linear function of the coordinates 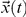 and their derivatives, system identification is difficult due to a lack of coherent and well defined theory for appraising such computations. When the upper limb dynamics is described, we expect the solution of equation (1) to be limited, non-chaotic and quasi-periodic. With these premises, the non-linear system (1) can be approximated with a time-varying locally linear system and can be recast in the following polynomial form [34]:
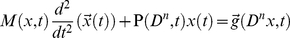
(2)where:
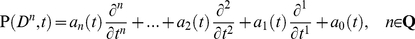
(3)is a polynomial operator[35].

Equation (2) is a model for a time-variant linear system whose oscillating solutions can be found both in the time and frequency domains by means of classical control theory. Assuming the system is stationary (i.e. 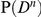 does not depend upon time and its coefficients 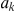 are constant), and under-damped, the measured angular frequencies 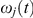 in response to a perturbation of the mechanical system (called resonant angular frequencies) are constant. Thus, classical Laplace transform techniques can be used to approach the problem in the frequency domain where equation (2) is recast in the form:
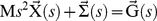
(4)The resonant frequencies are represented by the peaks on the absolute value of the complex spectrum of the solution of (4). If the system is second order, modal analysis of vibrating systems offers a variety of techniques to identify the characteristics of 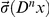 from the values of the constant resonant frequencies 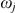. Specifically, coefficients 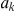 of 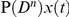 can be identified. When the system is linear but not stationary (i.e. the coefficients 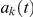 are a function of time), the frequency response following an impulsive perturbation will vary as a function of time. In this condition, equation (4) cannot track the time varying resonant frequencies and a new approach must be taken to identify 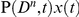. We achieved this by adopting the variation of the joint angle 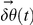 as the independent coordinate for our analysis. The solutions of equation (2) for the 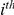 degree of freedom can then be expressed in terms of instantaneous amplitude and phase [29]:
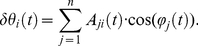
(5)where, 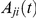 is the instantaneous amplitude for the 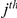 resonant frequency associated with the 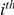 degree of freedom and 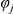 is the instantaneous phase. The 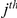 instantaneous resonant (or damped) angular frequency of the system is defined as the derivative with respect to time of the 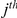 instantaneous phase:
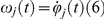
We present a technique to measure vectors of instantaneous resonant angular frequencies 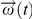 and instantaneous amplitude 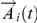, for the time-varying dynamics of a two degree-of-freedom double-pendulum system during the free response to an impulsive perturbation. The system models the human upper limb, during either postural or reaching tasks. When 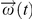 and 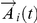 are known, and the system is second order and locally linear, modal analysis can be applied at each instant to reconstruct the characteristics of the internal force field 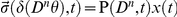.

### The Spectrogram

The convolution of window function 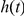 sliding along the non-stationary time-variant signal 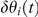 as a function of time shift 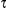 is called a “Short Term Fourier Transform” (STFT) and can be expressed as:
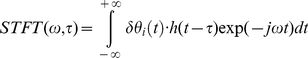
(7)


A spectrogram is the representation of a STFT calculated on the signal 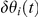 for multiple time shifts 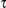 and is the tool used in the implementation of our time-frequency analysis. The value of the spectrogram at each instant is calculated as the average of all STFTs enclosing that instant in their respective window functions. Therefore, the peaks of the STFT spectrum at each instant represent the solution of the eigen-problem represented by equation (4) in the frequency domain at each time lag 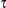. The spectrogram can be seen as a “complex energy density” distributed in time and frequency. This representation of energy density is “smeared” across all the windows encompassing a certain instant due to the averaging operation. To overcome this limitation, a representation of the STFT known as reassigned spectrogram (RS) can be used [36]. Since the STFT spectrum is a complex function of two variables (i.e. time and frequency) its maxima can be computed either by locating the points at which the Hessian (i.e. the matrix of second order partial derivatives with respect to time and frequency) of the function magnitude is zero, or by identifying the stationary points of the phase. The Hessian-based technique is unreliable since the smearing in frequency produces a wide plateau in the neighborhood of the maximal energy, limiting the resolution of the instantaneous frequency estimate. However, calculating the partial derivatives of the phase with respect to time and frequency identifies points of stationarity, and the associated time delay and a frequency shift that can be used to “reassign” the position of maximum energy [37]. RS methods, based on this re-mapping algorithm, can then provide a “super-resolution” in both time and frequency compared to traditional STFT [36]. However, the super-resolution cannot be constant throughout the frequency and time domain (locality) because of its dependency on the amount of smearing of the energy caused by the convolving windows [38], [39].

The RS transformation is always possible even when the system is in the form of equation (1) rather than equation (2). Standard modal analysis can be applied only if the system is locally linear and second order. However, if the system is higher than second order or weakly non-linear (without bifurcations, jumps, and chaotic behavior) we can still characterize “non-parametrically” the characteristics of the internal force field 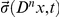 through the RS. The result represents a generalized force curve as a function of the positional modal coordinates [40].

### Assumptions and possible relaxations

In this section we describe the mechanical models we used to simulate the reaching movement of a human arm, and discuss the characteristics of each model. The assumptions under which our method operates are also discussed.

### System characteristics

**Figure 1:**
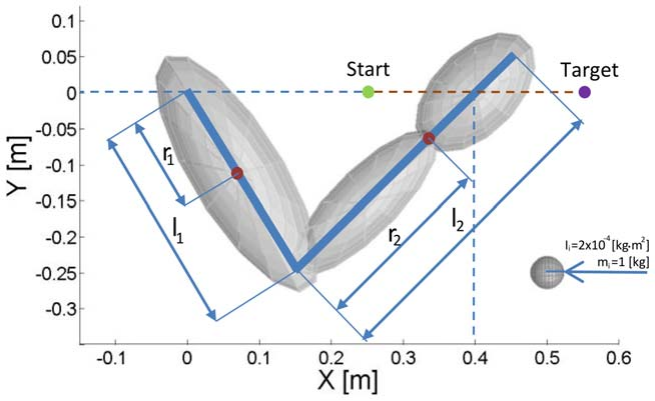
Representation of the double-pendulum model of the arm. The centers of the inertial ellipsoids represented in the figure are located at the centers of mass of the body segments. The length of the upper arm is 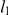, and the center of mass is at 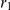 from the shoulder center of rotation. Hand and forearm are considered as a unit of length 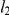 with no joint at the wrist. The resulting center of mass for the segment is obtained by the combination of those of the hand and forearm and is located at 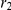 from the elbow. The size of each ellipsoid depends on both mass and inertial tensor of the segment. The dimensions of each ellipsoid along the major and minor axes (eigenvectors) are computed as 
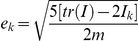
, where 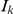 are the principal moments of inertia of the tensor 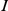, and 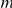 is the mass of the segment. During simulated movements, the hand's center of mass follows the trajectory shown as the dashed brown line. In the figure the hand center of mass is at position (0.4,0)m, which is the configuration used for the postural tests.

When we consider the rigid motion of a double pendulum as represented in Figure 1, the torques at the joints can be represented by the dynamic equation:
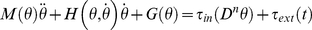
(8)where 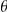 is the vector of joint angles, and 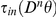 is the vector of muscle generated torques, which is a function of the joint angles and their derivatives. If along the movement trajectory, the subject is required to apply a force while still maintaining the desired trajectory, (e.g. pushing-pulling in a specific direction) the muscles will generate the additional torques 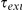, which are equivalent in magnitude to the torques generated by the external force acting on the limb. The vector 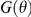 is the contribution of gravity to joint torque which is null when the gravity field acts orthogonally to the trajectory as in a horizontal, planar movement.

The inertial and Coriolis matrices 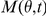 and 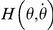 are in the form [41]:
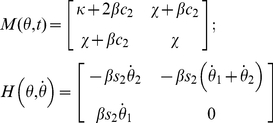
(9)where
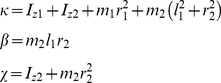
(10)


Subscript “i = 1” refers to variables of the upper-arm link and shoulder joint, and subscript “i = 2” identifies forearm-hand link and elbow joint variables. 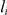 is the length of the 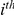 link; 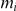 is its mass, 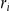 is the distance between the 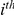 link center of mass and the 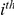 joint, and 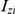 are the moments of inertia about the z-axis orthogonal to the plane of movement calculated at the 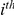 link's center of mass. We use simplified notation for trigonometric functions with 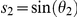 and 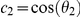.

A variational analysis of the torque generated as a variation of the trajectory is used to find the internal force fields exerted in response to a mechanical perturbation 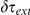. This is obtained by calculating the total derivative of equation (8) after moving 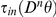 to the first member of the equation:
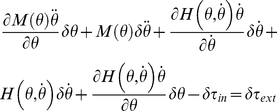
(11)It is convenient to define the system's internal force field so that:
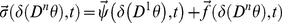
(12)where 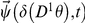 is the internal force field generated by the mechanism's dynamics, which includes the contributions of the derivatives of the Coriolis and centripetal forces with respect to the coordinates 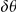 [15], [17], and 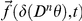 is the internal viscoelastic force field generated by the mechanical network, excluding the mass:
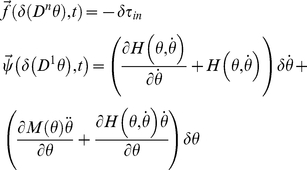
(13)


When the inertial parameters in (10) are known, 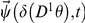 can be immediately calculated, independently of the viscoelastic characteristics of the system 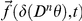.

Equation (11) can be recast in the form of equation (1) by substitution of equations (12) and (13). Defining 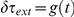 and the generalized coordinate as the variation of joint angle 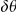 we obtain:
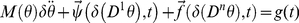
(14)


**Figure 2:**
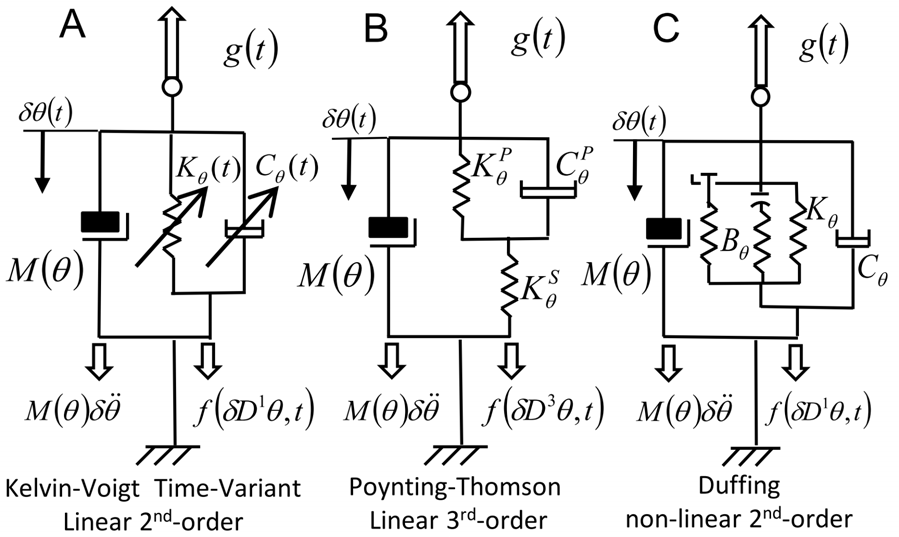
Mechanical models used in the simulations. A) Time-variant second-order viscoelastic linear system (Kelvin-Voigt). B) third-order viscoelastic linear system (Poynting-Thomson). C) Time-invariant second-order cubic viscoelastic system (Duffing). The schematics highlight the different force fields of the D'Alembert equation (2) when the internal forces generated by the dynamics are negligible. In the figure, each force field is dependent to the mechanical elements that generate it.

We will now analyze the time-frequency responses of three viscoelastic mechanical networks with oscillating behaviors. The schematic of each model is presented in Figure 2 as a single degree-of freedom (DOF) representation. It is also important to notice that exact tracking of the arm's unperturbed trajectory is not strictly necessary because the parameters are estimated in the frequency domain.

The system depicted in Figure 2a is commonly known as the Kelvin-Voigt (KV) model and is widely use to represent the mechanical behavior of the upper limb. A KV mechanical model is linear and second order, which allows us to use instantaneous modal analysis for the identification of system parameters under several combinations of stiffness and damping time profiles. The system internal viscoelastic force field 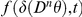 is represented by the differential equation:
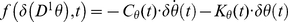
(15)


Most identification techniques proposed in the literature assume the damping 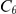 and stiffness 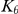 to be time-invariant. Our work relaxes this assumption by considering the coefficients as time-varying.

Figure 2b represents a linear, time-invariant, third-order system known as the Poynting-Thomson (PT) model. This mechanical network is an extension of the KV model commonly used in muscle models and includes tendon elasticity (Hill-type passive model). The PT model includes two separate elastic elements. The element 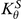, in series with the muscle fibers, represents the stiffness of the tendon. The parallel between 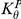 and 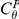 represents the stiffness and viscosity of the muscle fibers. The internal viscoelatic field complies with the following differential equation:
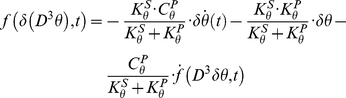
(16)


Figure 2c represents a non-linear system known in the engineering literature as the Duffing model. It provides an approximation of a tendon's slack behavior. Here, the stiffness depends on position, and is low for small displacements (slacking of the tendon) and increases abruptly after a fixed threshold. As a first approximation the stiffness of the model is considered to increase cubically (hardening system) which is compatible with experimental evidence found in human triceps surae muscle [42]. In general, Duffing type models can generate chaotic responses; however, we will restrict our study to a system with known stable behavior. In the time domain, Duffing viscoelastic force can be expressed using the differential equation [43], [44]:
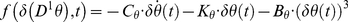
(17)The generalization to multiple DOFs is easily accomplished, and results in the parameters 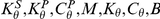 which can be expressed as matrices.

Since modal analysis cannot be used to identify parameters of PT and Duffing systems, we used the RS technique to identify non-parametrically the force characteristics of these systems.

#### Oscillatory behavior

We simulated the mechanics of a human arm with two coupled degrees of freedom (Figure 2) and estimated its response to a perturbation using the mechanical models in equations (15–17). The technique proposed here requires eliciting an oscillatory response by delivering a mechanical disturbance to the system. Measurable post-perturbation oscillations in free space indicate that the arm is an under-damped mechanical system. Postural measurements and single joint movement measurements [8], [45] also show the damping to be under-critical. The PT model is physiologically consistent with muscle-tendon systems and is often used as a linear, time-invariant approximation. A PT system exhibits one oscillatory mode, independently of the value of the muscle damping 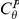, if 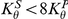 where 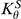 is the stiffness of the tendon and 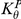 is the stiffness of the muscle fibers. An analytical proof is presented in Supplement S3. The under-damped PT model is third-order [46] and has one zero, one real pole, and one complex pole pair (see Supplement S4). When approximating PT as a second-order system (i.e. 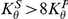), oscillating behavior is still assumed because the complex pole pair must be dominant (if the real pole were dominant the approximation would be a first-order system). The approximation to a second-order oscillating system is accurate when the zero and the single pole have similar values and their effects cancel out. The double pole dominancy with respect to both the zero and single pole is supported by stochastic non-parametric identification [5], [47], [48]. Given the ability to approximate the arm as a second-order mechanical system, the majority of the analysis described here will concentrate on parameter estimation for KV-type models. We did then generalize the findings to the more complex PT and Duffing models.

#### Separability

We assumed the two instantaneous resonant frequencies of the system (i.e. the peaks of the spectrogram as a function of time) to be distinct within the resolution limit of each transfer function spectrum. The representation of an unperturbed movement in the time-frequency domain resembles a function with a constant value at a frequency the inverse of movement duration. Impulse perturbations in the time domain appear in the frequency domain as instantaneous excitations of the entire frequency spectrum. The characteristic frequency of the movement is present in the spectrum before and after the impulse, while the frequencies proper of the oscillatory system are evident only at time instances following the perturbation. In practical terms, if a two degree-of-freedom system, such as the human arm in our model, is analyzed, the spectrogram shows one constant frequency before and after the impulsive perturbation and two additional frequencies after the impulse, making it possible to distinguish which frequencies are intrinsic oscillations of the system and which is a property of the movement. If the baseline movement has a long duration, the frequency of the movement will be lower than both the oscillatory frequencies of the system, and a high pass filter can be used to eliminate the movement frequency from the spectrum. When the lower vibrational frequency coincides with the frequency of the movement, the spectrum of the two oscillatory modes can be isolated from that of the movement by subtracting the time-frequency signal recorded prior to the perturbation from the signal after the perturbation. Signatures of the oscillatory properties of the robotic manipulandum, if present, can be similarly eliminated by frequency segregation.

#### Symmetry

When estimating the parameters of a KV system during posture, the assumption of symmetry of the stiffness matrix has been a controversial issue in the literature. Most studies of human arm stiffness indicate that the system is mostly conservative, or symmetric [4], [8], [10], [49], [50]. Asymmetry can be quantified by using the curl of the elastic field, which is directly related to the amount of energy that is dissipated by the system to make the hand follow a close trajectory in a non-conservative field [7]. Mussa-Ivaldi et.al. [7] demonstrated that for most subjects, the curl was present but not statistically significant, and when significant, it accounted for a restoring force that was much smaller than the spring-like behavior. Dolan et al. [6] obtained asymmetric stiffness matrices where the curl was on average 25% or smaller in most of their subjects. However, no statistical analyses were performed on the curl statistical significance. Given the estimation uncertainty of each stiffness coefficient, it cannot be ruled out that the off-diagonal terms would represent the same values within the uncertainty interval. The assumption of symmetry might not apply to all double joint sets. Lacquaniti and colleagues [51], obtained a highly asymmetric joint stiffness estimation for the elbow-wrist joint couple. However, the estimation was carried out around a configuration representing a singularity for the Jacobian matrix (hand outstretched). The singularity of the Jacobian can induce singularities in the stiffness matrix, thus compromising the assumption of a conservative elastic field [52].

During movements, statistically significant asymmetries of the stiffness matrix were also reported by Franklin et al. [12], [13] using the estimation method proposed by Burdet et al. [11]. This method did not require a calculation of inertial parameters. Instead, stiffness was estimated independently from other mechanical components by applying a “ramp and hold” perturbation on a predicted endpoint trajectory through the use of a stiff robotic manipulandum. A steady state displacement was reached at the end of the perturbation, where the variation with respect to the unperturbed trajectory of both velocity and acceleration was negligible. To understand why such dynamic stiffness measures can exhibit asymmetries, recall equations (11–14), and consider the internal viscoelastic field 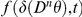 of a KV model such as in equation (15):
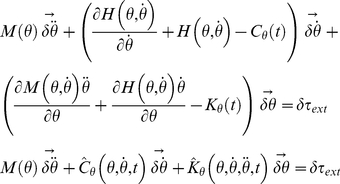
(18)


To transform the stiffness from the Cartesian space to the joint space, the following kinetostatic equation applies :
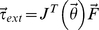
(19)Where 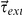 is the torque at the joints necessary to generate the force 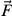 at the hand, and 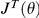 is the transposed Jacobian matrix, which is a function of the joint angles 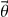. Knowing that the Cartesian stiffness is 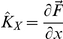, the Jacobian matrix is 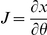, and from equation (18) that 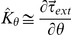, we can write the derivative of the kinetostatic equation with respect to the Cartesian coordinates 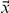 so that:
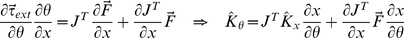
(20)It follows that :
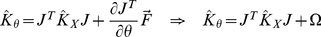
(21)and finally
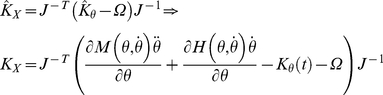
(22)


When the unperturbed reaching trajectory can be provided as a baseline, the displacement that results from applying a displacement perturbation 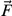 is small, and the matrix 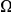 is negligible. Furthermore, in all experiments based on the same technique presented by Burdet et al. [11], the effect of 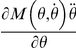 was small because the stiffness estimation was usually performed at the middle point of a reaching movement, where the angular acceleration 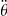 was close to zero. However, the term 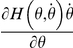 could be non-negligible because the joint angular velocity 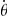 would be maximal in the middle of the movement. The matrix 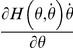 is non-symmetric (Supplement S1), and might be responsible for some of the asymmetry reported using the technique of Burdet and colleagues [11].

#### Complex modes

The estimation of a system's stiffness and damping parameters by analysis of its oscillatory modes requires the solution of an eigenproblem: the eigenvalues and the eigenvectors of the viscoelastic force field 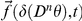 must be estimated. Either proportional or classical damping is often assumed [53] and these two conditions impose a constraint on the viscous component of the viscoelastic field 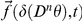. Proportional damping assumes the viscous field component to have a magnitude that scales linearly with the elastic field component. Classical damping considers the viscous field to be aligned with the elastic field, but does not impose constraints on its magnitude. A necessary and sufficient condition for a system to be classically damped is that the eigenvector of the internal viscous field must be aligned to the eigenvectors of the elastic field [54]. In a second-order system, eigenvectors identify the axis of the stiffness and damping ellipses. Although Frolov and collegues [14] found the stiffness and damping ellipses to be similarly oriented, considerable variability existed.

Our approach requires no a priori assumptions about damping parameters besides symmetry and as we will show, it can identify the system parameters even in the presence of a misalignment between the damping and stiffness eigenvectors by allowing for “complex modes” [55] when solving the eigenproblem. Moreover, we will demonstrate that the estimation of stiffness with our technique is minimally influenced by the value of damping parameters within the ranges commonly reported in the literature.

### Equation normalization

Using a planar two degree-of-freedom model of the arm, inertial and anthropometrical parameters in equation (10) were calculated from a single averagely built “subject” (see Table 1). Nine commonly used sets of regressive equations were implemented: Hanavan (HV) [56], Dempster (DE) [57], Chandler (CH) [58], Clauser (CL) [59],McConville (MC) [60], Zatsiorsky and Seluyanov (Z1) [61], Piovesan (PI) [41], Zatsiorsky and Seluyanov (Z2) [62] and de Leva (DL) [63]. Inertial parameters were computed with each of these nine inertial models to allow a sensitivity analysis (see “Results”). We used the method described by Zatsiorsky [62] as a reference standard because we had found earlier [41] that this method best approximates the true inertial parameters across the aforementioned set of inertial models.

**Table 1:** Inertial and geometrical parameters used in the simulations.

Symbol	Denomination	Value
l_1_	Upper arm length	0.29 [m]
r_1_	Upper arm center of mass	0.132[m]
m_1_	Upper arm mass	1.99 [kg]
Iz_1_	Upper arm moment of inertia about the center of mass	0.0161 [kg m^2^]
l_2_	Forearm+hand length	0.4 [m]
r_2_	Forearm+hand center of mass	0.17 [m]
m_2_	Forearm+hand mass	1.10 [kg]
Iz_2_	Forearm+hand moment of inertia about the center of mass	0.0146 [kg m^2^]

Parameters were obtained from one subject using a regression equation proposed in [55].

The inertial matrix in equation (9) is real and positive definite and admits 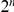 real square roots. Without loss of theoretical rigor, we can consider only its positive square root and define a new positive definite matrix 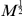 that is invertible [64]. The matrix 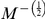 therefore exists and is symmetric and real. For a free response, the external field defined in equation (14) is 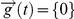 and we can normalize (14) by multiplying its first member by 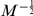, thus:
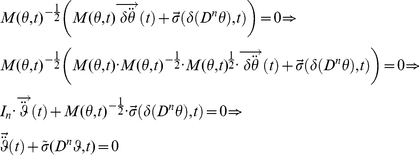
(23)where 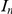 is the identity matrix for 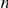 DOFs and 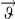 is a new set of normalized modal coordinates
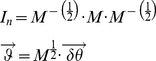
(24)The free response of a second-order KV system, as in equation (18b) can be described as:
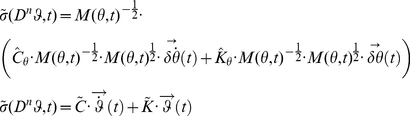
(25)where
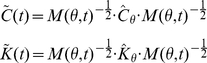
(26)


Substituting (25) in (23), equation (14) can be normalized using the inertial matrix to obtain a monic system, where spectral algebraic theory applies [65], [66], [67]:
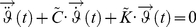
(27)



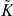
 is the normalized stiffness also called the “system matrix” or the “modal matrix”, 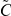 is the normalized damping matrix. The dependency of the normalized matrices on time and kinematics of the system has been omitted to simplify the notation. The normalized monic system (27) has the same eigenvalues as the original system (14) and eigenvectors dependent on the normalization. Note that, because of the properties of 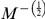, when 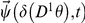 is negligible, the matrices 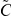 and 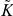 are symmetric and real [68].

### Identification of eigenvectors

We assume the system (2) to be underdamped, hence having 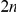 eigenvalues occurring in 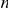 complex conjugate pairs, 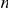 is the number of DOFs:
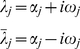
(28)where j = [1,2] for a two DOF system. In the general case of non-classically damped system, if 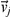 is the eigenvector associated with 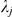, the corresponding eigenvector of 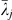 will simply be the complex conjugate 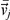 [55]. A linear combination of the eigen-solutions represents a general solution to (2):
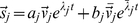
(29)


If the system is classically damped, all the eigenvectors of the system will be real [55], [69], so that:
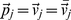
(30)and the matrix of the system eigenvectors can be written as:
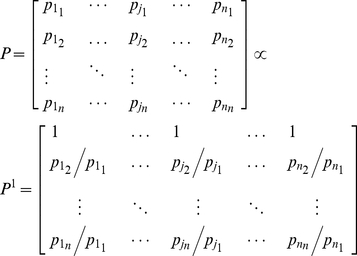
(31)


In 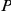 the magnitude of each vector is normalized to 1, and in 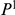 the first component of the vector is normalized to 1.

To be a physically possible solution, each 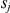 in equation (29) must be real, hence 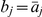 [55], therefore:
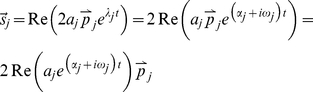
(32)In general 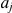 is a complex number and it can be written in the exponential form 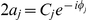, with 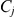 and 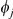 real [55].

After substitution, (32) can be written as:
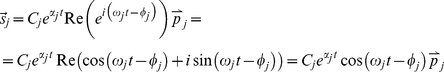
(33)


The general solution of (2), or the linear combination of all the solutions of the eigenproblem, can be interpreted as the super-position of each damped mode of vibration [55], and in general can be written in the form:
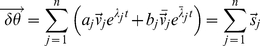
(34)


Since (2) is not decoupled, the free time response of each degree of freedom will be of the form (34). If the instantaneous reassigned frequencies are sufficiently far apart from each other (separable in the frequency domain), then each independent damped mode can be isolated at each instant using a filtering process. Each 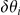 is high-pass and low-pass filtered within a sliding window 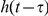, at a cutoff frequency located at the average between adjacent instantaneous frequencies derived from the RS within the same window. In our case (a two DOF system), using (33) and (34) we obtain: 
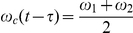
. For convenience the window and the hop size are the same as those used for computing the spectrogram.
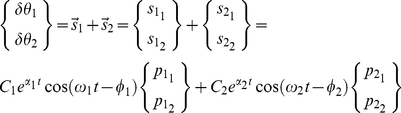
(35)Recalling (5) we can see that
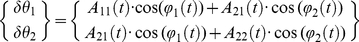
(36)and from (35) and (36) that
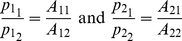
(37)


Each time-varying eigenvector in 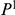 can be calculated as the ratio between the instantaneous amplitude of each modal coordinate's mode.

If the system has complex modes, the eigenvectors of the system will be complex and can be represented in the form:
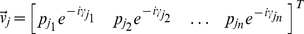
(38)


Substituting (38) in (33), each mode can assume the following general form:
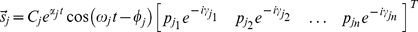
(39)


A physical interpretation of this formulation is that the 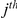 mode oscillates with frequency 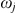 and decays with a damping ratio 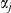, and each of its 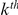 components presents a phase shift of 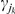.

In the case of a two DOF system, equation (39) can be written as:
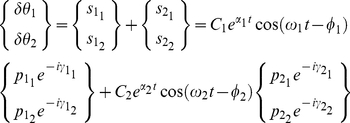
(40)


It follows from (40) that:
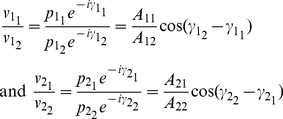
(41)


The difference in phase between 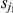 and 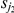 is then 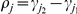 [40]. Because 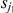 and 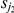 are time signals with the same frequency, the time lag between the two is equal to:
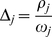
(42)

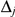
 can be found using a cross-correlation function between the components of each mode characterized by the same frequency.

For a 2 DOF system, when 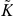 and 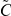 are symmetric, 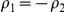, we will show that 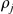 is equal to half the rotation of the damping matrix eigenvectors with respect to the stiffness matrix eigenvectors. If the system is assumed to be non-symmetric, each 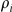 should be identified independently.

### System Decoupling and Modal Analysis

The signals 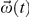 and 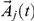 are related to the coefficients that decouple equation (2). Assuming the system linear and second order, the values of the matrices 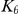 and 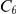, representing stiffness and damping respectively, can be estimated from the decoupled system (eigenproblem solution) under the hypothesis of an under-damped mechanism with known inertial parameters (“inverse problem”). The solution of the inverse problem requires that the eigenvectors and eigenvalues of the system be known. While the eigenvalues can easily be obtained from a spectrogram since they uniquely represent the resonant frequencies of the system, the eigenvector (i.e. the modes of vibration) must be reconstructed from the measured data in a convenient modal reference frame. In a symmetric classically damped system, the eigenvectors for both the normalized stiffness and normalized damping in (27) are the same, and can be reconstructed from the instantaneous amplitude of the spectrogram. In a non-classically damped system, a further step is necessary to estimate the phase difference 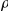 between the modes. Once the matrix of eigenvectors 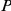 is estimated we can use its properties to decouple the normalized system (27) so that:
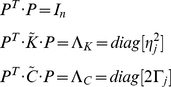
(43)where 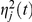 is the eigenvalue of 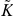 which corresponds to the 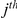 squared “natural” or “undamped” angular frequency, and 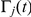 is the eigenvalue of 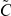 corresponding to the 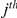 universal damping ratio. Therefore, equation (27) can be rewritten as follows:
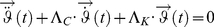
(44)


If the instantaneous “resonant” angular frequencies 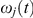 are not constant, then the normalized squared “natural” angular frequency 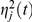 and the universal damping ratio 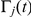 associated to each of the 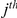 vibrational modes are time-varying and can be estimated as follows [40], [70]:
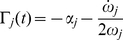
(45)

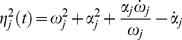
(46)where
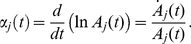
(47)If the system is second-order, by knowing the matrix 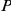 we can reconstruct (27) from (44), and by having defined 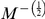 we can compute (2) from (27), obtaining an estimation of the stiffness 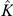 and damping 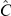 in the time domain, namely:
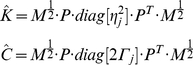
(48)Furthermore, by knowing 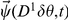, 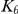 and 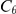 can be readily estimated from (18).

The parametric modal analysis here described cannot be applied to Duffing or PT models. However, spectral decomposition is still possible given the oscillatory behavior of the system. Hence it is still possible to identify the instantaneous resonant frequency 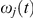 and amplitude 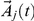 for each degree of freedom. Equations (45–47) still apply, therefore we can estimate the features of the internal force [71].
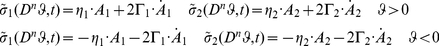
(49)


### Description of the Simulation

A planar two degree-of-freedom model of the arm was used to analyze both static postural and reaching movement conditions (Figure 1). The model was implemented using Simulink® (The Mathworks®, Natick, MA). During simulations of arm movement, the center of mass of the hand followed an imposed straight trajectory on the horizontal plane, parallel to the sagittal plane. The origin of the reference system was placed at the center of rotation of the shoulder with 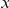 axis parallel to the direction of movement and positive distally and the 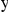 axis positive medially. The starting position at t = 0 was at a point (0.25,0)m in front of the shoulder, and the target was located at point (0.55,0)m (Figure 1).

The hand trajectory varied sigmoidally in time described by the equation:
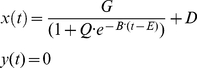
(50)where
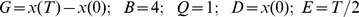
(51)


**Figure 3:**
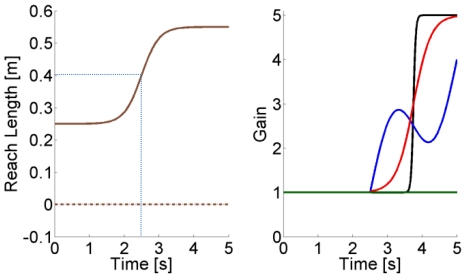
Representation of the imposed reaching trajectory and the multipliers for the stiffness time profiles. In the left panel, the reaching profile for the x (solid) and y (dashed dotted) components of movement are represented using the convention of Figure 1. The co-ordinates shown in light blue refer to the position of the hand's center of mass used in the static (postural) condition. For the first part of the trajectory, a constant stiffness and damping are imposed at the beginning of the movement (right panel). Subsequently, after the application of a force impulse perturbation, the joint stiffness is modulated by means of the gain profiles depicted on the right panel. We imposed a constant (green), slow sigmoidal (red), a combination of linear and sinusoidal (blue), and sharp sigmoidal gain (black), respectively. The same time-varying profiles are also imposed to stiffness and damping during the simulated static condition.

We used 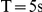 of simulated time, with sampling at 4 kHz, to allow the sigmoid to start with zero curvature. Effective movement duration was 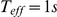, defined as the time between 10 and 90% of the total amplitude 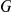 (Figure 3a). Dynamic stiffness was tested between (0.4,0)m and (0.55,0)m in the second half of the trajectory, between time T = 2.5 s and T = 5 s during the movement. Postural time-varying stiffness was tested in the same time interval with the hand at point (0.4,0)m which corresponded to the center of the simulated reach.

#### Time-variant Kelvin-Voigt System

For both the postural and the movement simulated paradigms, the reference joint stiffness and damping were set at
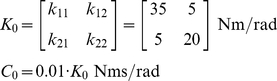
(52)where 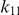 is the stiffness of the shoulder joint, 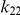 is the stiffness of the elbow joint variables, and 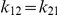 is the intra-joint stiffness. The values chosen for the simulation match published values for stiffness during multijoint movements [11], [15], and for damping during postural and single joint movements [6], [8], [45]. We tested three separate time-varying stiffness and damping profiles for the movement and postural cases by modulating the reference values of the parameters. Different profiles were implemented by multiplying all coefficients of either the stiffness or damping matrix by one of the following time-profiles 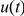: a “constant”, a slow varying “sigmoid”, the sum of a ramp and a sinusoid (“sinlin”), and a “sharp” varying sigmoid (Figure 3b):
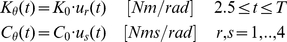
(53)where
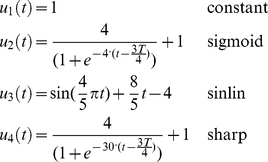
(54)


Finding the stiffness at the beginning of a movement is an important goal in motor-control and might help in shedding some light on how a forward model may evolve as adaptation occurs [72]. However, the velocity of the hand at the beginning of a movement is low, so that the arm kinematics might not clearly differ from the postural task. Hence, to maximize the differences in joint velocity between the postural and the movement cases, we simulated variations of the stiffness profiles starting at the mid-point of the movement where the reaching speed is maximal. This allowed us to test the sensitivity of the measure at the same position but at two very different speeds, hence obtaining a wider interval of validation. The analysis during movement presented here has been validated from the middle of the movement to well after the movement's end. The same estimates can be made 50 ms after the onset of a movement (where velocity is also low), by applying a brief impulse (20 ms) about 30 ms after movement onset. This procedure can be applied throughout the workspace because the technique is largely insensitive to the configuration of the arm.

The stiffness was constant at the start of each condition, and began to change at time 2.5 s. At the same instant, a brief force perturbation (5 N for 20 ms) was applied to the hand in a direction chosen randomly among the eight octants of the horizontal plane. The perturbation characteristics were based on the bandwidth that such an impulse excites (the shorter the impulse, the wider the band) and the amount of momentum that can be injected to introduce an oscillation big enough to be detected but small enough to not disturb the intended trajectory. Ideally, such a perturbation might go unnoticed if it induces a deviation from the planned trajectory that is near the level of motor-noise, thus allowing the subject to complete the intended movement without voluntary corrections [73]. The system will resonate with the same frequency and modes independently of the direction chosen if the impulse excites a bandwidth containing all resonant frequencies [74]. The estimation of stiffness of a real arm might depend upon impulse direction if reflexes of different muscles are excited depending on the perturbation direction. In that case more than one perturbation might be necessary to assess the average behavior.

**Figure 4:**
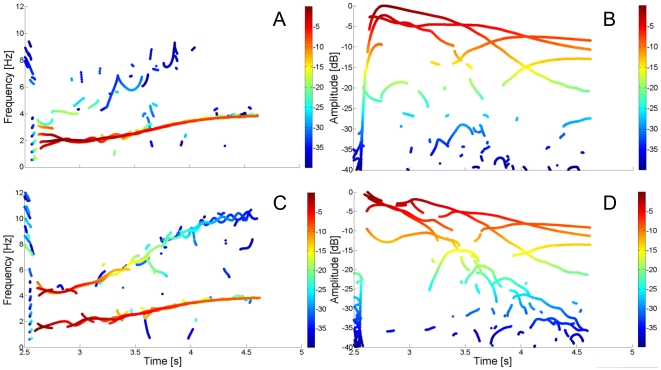
Orthogonal projection of the reassigned spectrogram for each separate joint. Orthogonal projection of the reassigned spectrogram for the variables 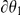 (A,B) and 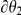 (C,D) calculated with the maximum noise level (SNR = 10 dB). Due to the different orientation of the eigenvector matrix 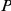, the second frequency of 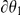 (A,B) has a lower power compared to that calculated for 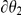 (C,D); hence, the oscillation is still present but it is just above the noise level. The estimation of instantaneous frequency 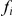 and instantaneous amplitude 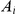 are however very clear when analyzing the spectrogram of 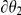 (C,D).

The STFT spectrogram was calculated using a 0.75 s Kaiser window, with β = 3. Convolving the window every 2.5 ms (hop size), provided a base resolution of 1.33 Hz and 0.0025 s in frequency and time, respectively. A higher resolution in frequency was achieved by calculating the RS with the same parameters. A third order Savitzky-Golay polynomial filter [75] with a 0.25 s window was used to obtain a continuous function of instantaneous angular frequencies 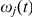 and amplitudes 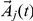 (see Figures 4–6),

**Figure 5 pone-0033086-g005:**
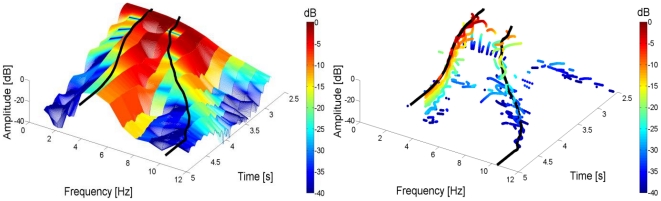
Example of Spectrograms. Short Time Fourier Transform (STFT) Spectrogram on the left and Reassigned Spectrogram (RS) on the right for a simulated arm reaching movement with sigmoidal joint stiffness. Based on the classical spectrogram, the partial derivatives of the STFT phase with respect to time and frequency were calculated. This process identifies the location of the stationary phase with respect to the location of the window in the time and frequency domain. The time delay and frequency shift obtained with this process are then used to “reassign’ the position of maximum energy. Savitzky-Golay polynomial filtering allows for easy calculation of the RS peaks envelope. The envelope is depicted in both the classical and reassigned spectrogram in black. Note that it would be difficult to estimate accurately the peaks' envelope in the classical spectrogram due to the lower frequency accuracy.

**Figure 6:**
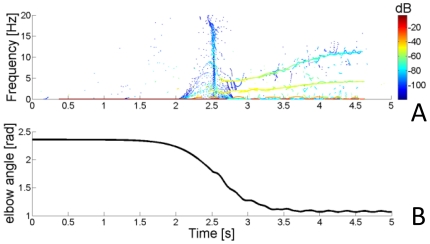
Frequency separation. A) Reassigned spectrogram of a perturbed movement. This panel illustrates the effect of an impulsive perturbation on the spectrogram of the elbow angular rotation, the frequency of the oscillations excited by the impulse are clearly identifiable. B) Time signal of the elbow rotation corresponding to the reassigned spectrogram in A).

To test the accuracy of the estimation techniques and the robustness to external disturbances and to non-repeatability of the subject's performance, the simulated hand position was corrupted with four levels of zero-mean Gaussian noise. Three levels of constant noise, with signal-to noise ratios (SNR) expressed in terms of the root mean square (RMS) of the signal, of 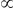 (i.e. no noise), 20 dB, and 10 dB, respectively. A fourth level of noise was signal dependent noise (SDN), proportional to the time-profile 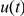 of the stiffness, scaling from no noise to a maximum of 10 dB. The SDN condition was used to simulate the assumption of proportionality of motor noise to muscle activation: the increase in joint stiffness can be attributed to an increased level of muscle co-activation [76], neglecting in first approximation the effects of reflexes and intrinsic stiffness. It follows that the higher the stiffness (and therefore the co-activation), the higher the level of noise disturbing the estimation 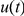 [77].

#### Non-Proportional Damping

In addition to the classically damped conditions presented in the previous section, we simulated non-classically damped systems. Taking 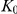 as a reference, we simulated a non-classically damped system by rotating 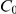 a specific angle 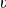, which resulted in a misalignment of the stiffness and damping eigenvectors, namely:
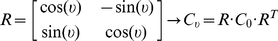
(55)


As an example we chose 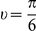. The components of the resulting modes presented a phase difference 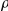 equal to half the rotation angle between the stiffness and damping matrices. The re-synchronization procedure described above produced a real eigenvector matrix 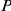 aligned with the eigenvector matrix of the normalized stiffness 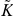.

We simulated the non-classically damped condition by implementing the sigmoidal time-profile 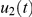 as a multiplier for the stiffness, and the “sinlin” profile 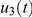 for the damping. SDN was added to the system.

#### Duffing System

Non-linear approximations to characterize limb mechanics often include a cubic stiffness term in addition to linear stiffness and damping terms [78], [79], [80], [81]. Maintaining constant stiffness and damping parameters 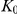 and 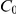 as in (52), we included a cubic stiffness term so that in the matrix version of (17) 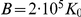.

#### Poynting-Thomson System

We chose parameters for the simulated PT model that were compatible with reported experimental measures [46]

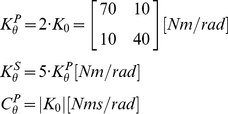
(56)All parameters in (56) were assumed to be constant during the simulated movements.

#### Regressive Techniques

We compared the results of our parameter estimations with those obtained by three well-known regressive techniques. Comparisons were carried out across all the conditions implemented on KV systems, including one regressive method based on force perturbations [14], [15] and two based on displacement perturbations [7], [8], [11], [17]. Displacement based techniques can be divided into those that estimate inertia, damping and stiffness (full regression) [7], [8], and ones estimating only the stiffness components (steady state regression) [11], [17]. For the force-based technique only, a full regression approach is applicable.

Following the methods of the respective papers, the force perturbation consisted of a 200 ms pulse with a 5 N magnitude, while the displacement perturbations were a deviation from the unperturbed trajectory with a maximum amplitude of 8 mm, lasting 300 ms (100 ms ramp-up, 100 ms plateau, 100 ms ramp-down about the unperturbed trajectory). To have the same number of points per estimate for both the force-based and displacement–based full regressions, only the first 200 ms of the displacement perturbations was used to compute the regression with the reaction force.

When full regression methods are used to estimate stiffness and damping during movements, even though inertial properties can be directly measured, they are usually evaluated in a separate static session to reduce the number of parameters to estimate at once [6], [15]. This approach is possible because inertial parameters are invariant with respect to the segments' centers of mass as seen in (10).

Methods that consider regressions at steady state [11] provide estimates of stiffness that are independent of the inertial parameters once particular conditions are met. As previously mentioned, estimating stiffness independently from the other mechanical components is possible toward the end of the perturbation plateau. In such a condition, if the robot is quite stiff, the variation with respect to the unperturbed trajectory of both velocity and acceleration is negligible, and the displacement reaches steady state. However, as seen in equation (18), this approximation might not be applicable at each point of the trajectory, especially if the stiffness is measured in positions with maximal acceleration. When we implemented this procedure in our simulations the last 50 ms of the plateau region was considered.

We estimated stiffness and damping at five different instants along the trajectory, starting at 2.5 s and then every 0.5 s. The actual location of each point of stiffness estimation depended on the methods specific to each technique. For each time-point estimation, one perturbation in eight different directions was used, resulting in a total of forty trials per method, for each of the four noise levels. We assumed the unperturbed trajectory to be known exactly. To compare directly the time discrete stiffness and damping profiles provided by each regressive method with the continuous estimation of the spectrogram method, we interpolated the punctual stiffness using a cubic Hermite spline. This method guaranteed a unique representation of each time-profile.

## Results

The parameter estimation of multiple stiffness and damping profiles carried out with our time-frequency technique described in the “Methods” section is compared to the identification of the same parameters with previously proposed regressive techniques. A non-parametric identification of higher than second order and non-linear systems is also provided.

### Identification of instantaneous frequencies and amplitudes

As implicit in equations (35) and (40), the time-frequency representation of the elicited vibrations 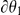 at the shoulder, and 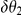 at the elbow, exhibit the same instantaneous frequencies 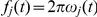, and amplitude decay 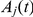 depending on the orientation of eigenvector matrix 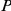, since the general free response to a perturbation is a superimposition of the two modes. This is evident in Figure 4 where the spectrograms of 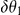 and 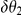 are depicted. The higher vibrational frequency is better defined in the spectrogram of 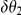. Since in the proposed simulated paradigm, the eigenvector component 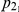 is small, so too is the energy content transferred from the perturbation to 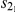 (the component of the 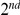 mode along the 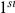 DOF).

Figure 5a, represents an example of a three dimensional view of the union between the spectrograms of 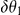 and 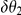. The regular STFT spectrogram representation and its reassignment can be compared. The RS enhances the resolution of the spectrogram and allows for a better identification of the instantaneous frequencies 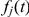, and amplitude decay 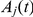, despite the presence of some easily identifiable computational artifacts. The figure also shows 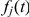 and 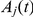 as functions of time, obtained with the polynomial filtering of the RS.

An example of an unfiltered reassigned spectrogram (RS) of a movement perturbed by an impulsive force is presented in Figure 6. In the time-frequency domain, an impulsive perturbation appears as a constant in the frequency domain (Figure 6a). This means that when an impulse is applied to a mechanical system, all the frequencies will be excited with approximately the same power. The instantaneous frequencies of the vibrational modes arise immediately after the impulse response. Figure 6b presents the time profile of elbow rotation 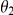 with the impulsive perturbation occurring at the movement middle point.

### Estimation of the Stiffness and Damping Matrices

**Figure 7:**
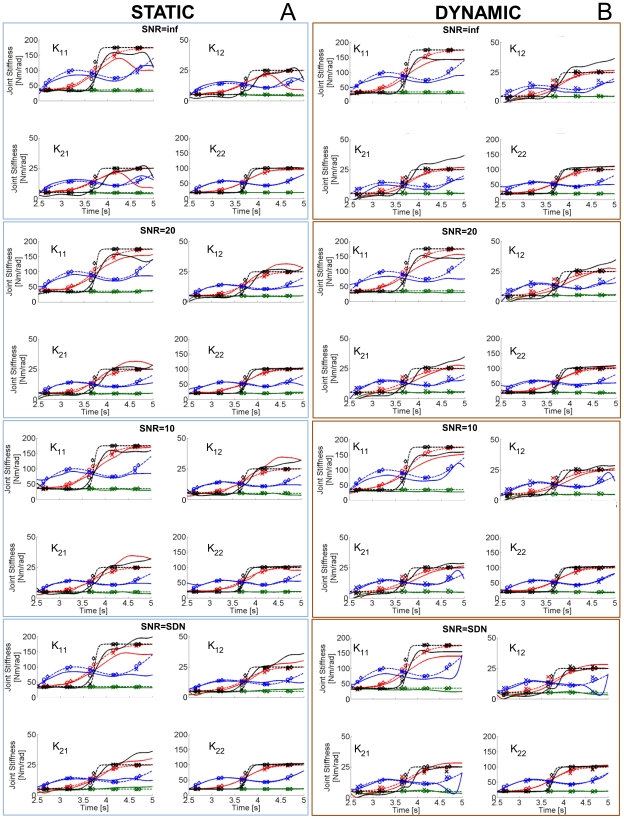
Stiffness estimation comparisons. A) Each graph represents the temporal variation of a specific component of the stiffness matrix as depicted in Figure 3. The hand is in a static posture at position (0.4,0) as represented in Figure 1. A “sinlin” damping profile is imposed and four profiles of stiffness are presented: constant (green), sigmoidal (red), sinlin (blue), sharp (black). Dashed stiffness profiles are those imposed in the simulation, while the solid-line profiles are the estimations obtained with the proposed spectrographic method. “X” represents the estimations of stiffness using a “full regression” from an imposed displacement. Each point represents the average stiffness within a 200 ms window.”◊” refers to the “steady state” estimations, notice that since the estimation is done at the end of the perturbation plateau, there is a time shift between “X” and “◊” of 75 ms. “O” represents the estimations using a full regression with an imposed force. Eight perturbations were applied to obtain each point of the stiffness with a regression. Only one impulsive perturbation was applied to obtain each full stiffness profile with the spectrogram technique. The different subpanels represent estimations of each element of the stiffness matrix with four different levels of noise. B) Equivalent estimations to those presented in A) but obtained during the movement condition, during which the hand's center of mass moves along the trajectory represented in Figure 3. Same nomenclature.

**Figure 8:**
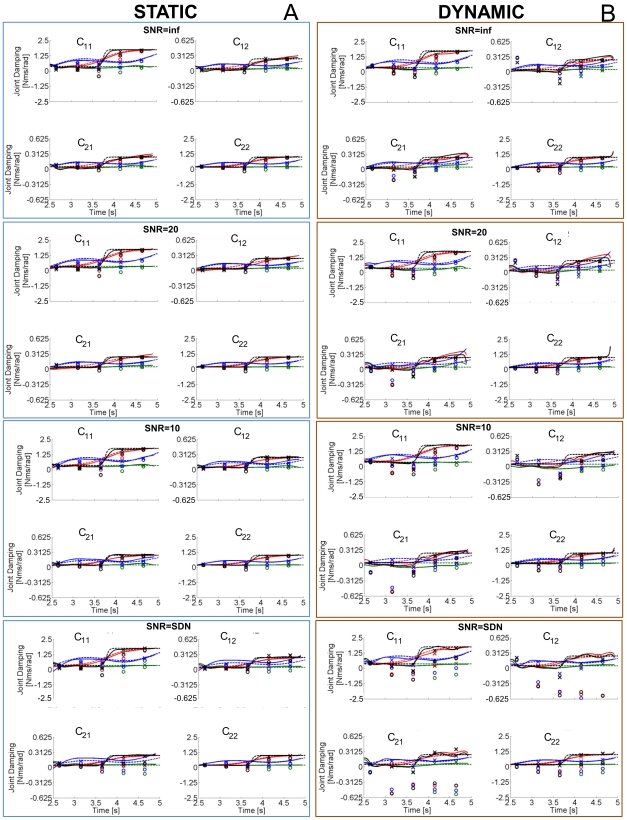
Damping estimations comparisons. Estimations equivalent to those in Figure 7 for the damping parameters, when a “sigmoidal” stiffness profile is imposed. The nomenclature is the same as in Figure 7.

To quantify the sensitivity of our method with respect to parameters of the mechanical model and to compare our results with those of published regressive techniques, we analyzed the performance of each method across a range of different parameter configurations. Stiffness and damping profiles were estimated in both static (postural) and dynamic conditions. As an example of the estimation, Figure 7 depicts the estimated stiffness profiles using the spectrogram and regressive methods when a “sinlin” damping profile is considered. Figure 8 presents all of the damping profiles when the stiffness changes sigmoidally. The estimation of stiffness matrix 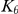 obtained with the modal analysis technique we propose is comparable to the result of regressive techniques, thanks to the small error in the estimation of instantaneous variables 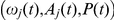 and the overall low susceptibility of our technique to noise.

Model performance is quantified by the percentage RMS error [82] of the fit compared to the stiffness or damping profile imposed during the simulation. As shown in previous work [82], using the percentage RMS error parameter provides a quantification of model performance under noisy conditions that is independent of the specific noise profile but is still dependent on the SNR. Interpolated stiffness and damping profiles (see ‘Regressive Techniques’) were used for calculating percentage RMS errors in the estimations based on regression.

One advantage of the method we propose, compared to regression based methods, is the ability to estimate continuous stiffness and damping profiles as a result of a single impulse perturbation. As explained in more detail in the discussion, the presence of damping in the mechanical system implies that the quality of the stiffness estimation is expected to degrade as the estimation instant becomes farther from the perturbation. However, it is possible to maximize the quality of the continuous estimation of stiffness and damping by utilizing perturbations with energy just high enough not to elicit voluntary corrections of the originally planned trajectory. A limitation of regressive techniques is that they can only provide punctuate estimations of stiffness and damping. The interpolation of the different punctual estimations along a time profile is theoretically unaffected by decay due to damping, and the percentage RMS error of the fit is expected to be low. However, multiple trials per estimation point, and multiple estimation points per time profile are required.

Obtaining comparable punctuate estimations of stiffness and damping using our method would be possible, provided that multiple runs of the simulations are executed under the same conditions, while imposing an impulse perturbation at a different position each time. However, such use of our method would defeat one of its inherent strengths, which is the ability to estimate stiffness and damping profiles during single movements.

So, to characterize our method locally, we chose also to quantify and compare different models' performance in terms of the percentage RMS error (E%) between 2.6 s and 3.175 s, which represents the interval between the first two instants following the perturbation at which estimations with regressive techniques are available. Even though the comparison window is limited, the interpolation on data obtained with regressive techniques requires more than two estimation points (each obtained regressing across many trials) to be acceptable, while our method provides the same data as a result of a single trial.

Percentage RMS errors were computed for simulated model fits across different estimation methods, imposed stiffness profiles, imposed damping profiles, noise levels, and static/dynamic conditions. An analysis of variance with repeated measures was carried out assuming the stiffness profile and the damping profiles to be random factors. The rationale for this choice is that during actual experimental use of the method we propose, the stiffness and damping profiles would not be known, and no assumptions about them should be required. The results of the ANOVA are presented in Table 2 (E% calculated along the whole available estimation interval) and Table 3 (E% calculated between 2.6–3.175 s). The data summarized in Table 3 are arranged to allow a pairwise comparison of all methods in Table 4. When the errors along the complete estimation interval are analyzed, all methods lead to statistically comparable results for all stiffness coefficients, across conditions (p>0.05). The pairwise comparison of the error on the first portion of the trajectory shows that our method produces estimates of the stiffness coefficients that are fully compatible with all three regressive methods (p>0.05 for estimate of all K coefficients). Interestingly, the same analysis shows that not all regressive methods produce statistically comparable results when implemented in our simulated tests. In particular, the estimation of off-diagonal elements of the stiffness matrix is statistically different between the full-force and full- displacement methods. In general, different methods do not produce comparable results in the estimation of damping coefficients. A complete set of tables that illustrates the error for each stiffness condition is included in Supplement S2.

**Table 2:** Repeated measures ANOVA among estimation methods with stiffness and damping time-profiles as random factors along the interval 2.5–5 s.

	*%E_k11_*	*%E_k12_*	*%E_k22_*	*%E_c11_*	*%E_c12_*	*%E_c22_*
Source	F	p	F	p	F	p	F	p	F	p	F	p
**method**	0.57	0.64	0.65	0.60	0.94	0.46	6.92	*0.013	8.86	*0.007	9.2	*0.004
**K Profile**	5.05	*0.025	7.99	*0.0046	3.91	*0.044	3.9	*0.04	1.77	0.23	2.29	0.14
**C Profile**	0.71	0.58	0.62	0.62	3.61	*0.033	2.76	0.08	1.93	0.23	1.69	0.23
**noise**	1.33	0.67	6.22	0.16	0.85	0.5	31.72	*0.0002	14.73	*0.003	27.49	*0.02
**condition**	16.53	*0.04	25.91	*0.014	3.98	0.13	9.91	*0.025	21.06	*0.008	18.13	*0.01

* Statistically significant parameters. Notice that the estimation method is not a statistically significant factor for the stiffness error.

**Table 3:** Repeated measures ANOVA among estimation methods with stiffness and damping time-profiles as random factors along the interval 2.6–3.175 s.

	*%E_k11_*	*%E_k12_*	*%E_k22_*	*%E_c11_*	*%E_c12_*	*%E_c22_*
Source	F	p	F	p	F	p	F	p	F	p	F	p
**method**	6.35	*0.012	13.84	*0.0005	0.26	0.85	7.13	*0.018	4.9	*0.042	5.84	*0.03
**K Profile**	0.12	0.94	1.43	0.28	2.01	0.15	1.56	0.29	0.56	0.65	1.92	0.2
**C Profile**	0.26	0.85	0.22	0.88	3.74	*0.03	3.69	0.06	1.8	0.31	0.91	0.47
**noise**	5.27	*0.028	1.24	0.37	0.93	0.46	29.06	*0.03	6.66	*0.027	22.79	0.051
**condition**	1.06	0.38	97.3	*0.015	19.97	*0.046	26.46	*0.025	24.58	*0.01	18.29	*0.009

* Statistically significant parameters.

**Table 4:** Pairwise repeated measures ANOVA between estimation methods with stiffness and damping time-profiles as random factors along the interval 2.6–3.175 s.

	*%E_k11_*	*%E_k12_*	*%E_k22_*	*%E_c11_*	*%E_c12_*	*%E_c22_*
Source	F	p	F	p	F	p	F	p	F	p	F	p
**Spectr.-vs-full disp.**	0.63	0.48	4.77	0.09	0.99	0.39	7.33	*0.04	13.05	*0.018	9.21	*0.025
**Spectr.-vs-full force**	6.72	0.075	0.41	0.56	1.68	0.27	6.05	0.07	7.07	*0.04	5.5	*0.075
**Spectr.-vs-SS disp.**	0.09	0.78	0.07	0.81	0.99	0.39	N/A	N/A	N/A	N/A	N/A	N/A
**full disp.-vs-full force**	0.32	0.61	22.9	*0.03	0.63	0.48	6.36	0.053	3.81	0.15	10	*0.02
**full disp.-vs-SS disp.**	0.98	0.4	0.64	0.48	0.96	0.4	N/A	N/A	N/A	N/A	N/A	N/A
**full force.-vs-SS disp.**	0.78	0.44	0.02	0.99	0.86	0.42	N/A	N/A	N/A	N/A	N/A	N/A

**Figure 9:**
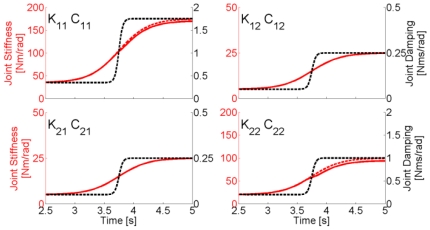
Effect of neglecting damping on stiffness estimation. Dashed lines represent the imposed stiffness (red), and damping (black) time-profiles, in accordance with the color-code of Figure 3. The solid lines represent the estimated values of stiffness coefficients when the natural frequencies of the system 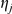 are assumed equal to the resonant frequencies 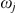 therefore neglecting the damping contributions 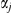 in equation (46).

* Statistically significant parameters. Only the influence of the methods is shown in the table. Spectrogram (Spectr), Displacement-based full regression (full disp.), Force-based full regression (full force.), Displacement-based steady-state regression (SS disp.),

To quantify the influence of different damping profiles on the estimation of stiffness, we performed a sensitivity analysis of the effect of damping on our method. According to equation (46), the effect of damping is to shift the natural angular frequency, hence affecting the estimation of stiffness. However, we found that by approximating the natural angular frequency 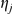 with the instantaneous resonant frequency 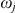 produces little effect in the estimation of stiffness as shown by the results in Table 5. An example of such an approximation is shown in Figure 9. The maximum RMS error of this approximation, when the stiffness varies sigmoidally, is 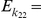
4.74% for a damping profile varying with a “sharp” profile. Notice that the approximation accuracy degrades along the trajectory as the damping increases (Figure 9). The value of damping coefficients reported in the literature [8], [23], [45] is generally small. To simulate a case in which the damping had a significant effect on the mechanical model, the imposed value of the damping coefficients necessary had to be about four times the average reported in [8] (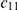 reaches 1.75 Nms/rad).

**Table 5:** Effect of neglecting damping on stiffness estimation with stiffness varying sigmoidally.

*C profile*	*%E_k11_*	*%E_k12_*	*%E_k22_*
const	0.26	0.006	0.56
sigmoid	1.83	0.04	3.92
sinlin	1.79	0.04	3.84
sharp	2.21	0.05	4.74

The spectrogram-based method proposed here requires a separate estimation of the inertial body segment parameters (BSP). BSPs can be obtained from models that apply to the geometry and the morphology of the subject. We investigated the sensitivity of the time-frequency technique to the nine inertial models proposed in [41], applied to the set of anthropometric measures described in Table 1. The same set of inertial parameters was imposed on the simulated system and used for stiffness and damping estimation. The inertial models of Hanavan (HV) and Dempster (DE), which performed poorly in the estimation of torque via inverse dynamic as discussed in [41], also produced less accurate estimations of stiffness and damping (see Supplement S2 for details) and were excluded from the statistical analysis. We also analyzed the sensitivity of the estimates produced by our method to the direction of the perturbation using eight perturbation directions, uniformly distributed in the Cartesian space along the octants. Table 6 shows the results of an analysis of variance with repeated measures of the percentage RMS error of the estimations, calculated with our technique across conditions and across inertial models. Damping profiles, stiffness profiles, and perturbation directions are assumed to be random factors in the analysis. The perturbation direction affected the estimation of 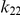 (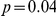), suggesting that some perturbations could align with one of the eigenvector of the system thus not properly exciting the resonant frequency of the elbow. Different inertial models, when tested across conditions, did not statistically affect the estimation of stiffness and damping coefficients. Out of the nine inertial models tested, the one proposed by Zastiorsky [55] provided the best compromise between percentage RMS errors in the estimations of stiffness and damping (Supplement S2), and therefore can be considered to be the best candidate for practical use with our method.

**Table 6:** Repeated measures ANOVA for the percentage RMS error using spectrogram technique among different inertial methods with directions of perturbation, stiffness and damping time-profiles as random factors along the interval 2.5–5 s.

	*%E_k11_*	*%E_k12_*	*%E_k22_*	*%E_c11_*	*%E_c12_*	*%E_c22_*
Parameters	F	p	F	p	F	p	F	p	F	p	F	p
**# stiffness**	2.30	0.13	3.58	0.05	1.42	0.27	1.01	0.55	1.34	0.30	1.85	0.18
**# damping**	1.94	0.27	1.28	0.42	3.03	0.16	2.26	0.20	1.46	0.37	1.31	0.40
**# direction**	0.44	0.84	1.68	0.18	2.50	*0.04	2.27	0.11	0.29	0.94	1.83	0.16
**inertia**	1.12	0.37	0.78	0.57	1.52	0.22	0.21	0.96	0.39	0.85	0.20	0.96
**noise**	2.01	0.17	0.04	0.99	1.72	0.18	2.99	0.26	0.63	0.60	3.13	0.05
**condition**	3.67	0.11	2.86	0.14	3.06	0.13	18.39	*0.01	5.81	0.09	15.52	*0.01

* Statistically significant parameters.

A sensitivity analysis compared how estimations of stiffness and damping RMS errors obtained by regressive techniques were affected by variations in system inertia, where again stiffness and damping were considered as random factors. Full regression techniques provide an estimation of the inertia matrix along with the stiffness and damping matrices and do not require a priori computation of the system inertia. Imposed variations of the inertia resulted in significant variations in the estimation of all stiffness and damping RMS errors, with the exclusion of coefficients 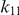 and 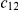 estimated with the displacement-based full regression, and coefficient 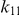 estimated with the force-based full regression, as shown by the results of the ANOVAs in Tables 7, 8, and 9. This result suggests that estimates of stiffness and damping with regressive techniques are affected by variations of inertial parameters of the limb even when such variations are within a physiologically plausible range. In particular, slight variations of arm configuration should be minimized in order to maintain consistent inertial parameters across trials.

**Table 7:** Repeated measures ANOVA for the percentage RMS error using force full regression among different inertial methods with stiffness and damping time-profiles as random factors along the interval 2.5–5 s.

	*%E_k11_*	*%E_k12_*	*%E_k22_*	*%E_c11_*	*%E_c12_*	*%E_c22_*
Parameters	F	p	F	p	F	p	F	p	F	p	F	p
**# stiffness**	363.15	*<0.0001	13.59	*0.03	1375.22	*<0.0001	9.80	*<0.0001	1.91	0.18	6.62	*0.02
**# damping**	2.64	0.13	0.69	0.67	1.91	0.32	6.08	*<0.0001	1.51	0.31	4.07	0.05
**inertia**	4.62	*0.01	5.67	*<0.0001	21.56	*<0.0001	27.14	*<0.0001	14.08	*<0.0001	20.53	*<0.0001
**noise**	7.92	0.88	0.50	0.69	33.85	*<0.0001	10.26	*<0.0001	6.42	*<0.0001	10.85	*<0.0001
**condition**	0.18	0.70	11.16	*0.04	15.84	*0.03	8.52	*0.03	23.28	*0.01	17.72	*0.01

* Statistically significant parameters.

**Table 8:** Repeated measures ANOVA for the percentage RMS error using displacement full regression among different inertial methods with stiffness and damping time-profiles as random factors along the interval 2.5–5 s.

	*%E_k11_*	*%E_k12_*	*%E_k22_*	*%E_c11_*	*%E_c12_*	*%E_c22_*
Parameters	F	p	F	p	F	p	F	p	F	p	F	p
**# stiffness**	26.77	*0.01	3.54	0.16	17.35	*0.01	3.12	0.14	1.20	0.42	4.78	*0.04
**# damping**	1.49	0.28	0.79	0.53	1.24	0.40	3.92	*0.05	2.39	0.21	4.96	*0.02
**inertia**	1.68	0.18	19.07	*<0.0001	10.21	*<0.0001	10.68	*<0.0001	2.55	*0.05	3.82	*0.01
**noise**	3.91	0.14	0.86	0.53	9.80	*<0.0001	48.77	0.90	1.91	0.26	19.86	0.25
**condition**	2.05	0.25	23.08	*0.02	7.33	0.07	1.72	0.26	11.33	*0.02	0.95	0.39

* Statistically significant parameters.

**Table 9:** Repeated measures ANOVA for the percentage RMS error using displacement steady state regression among different inertial methods with stiffness and damping time-profiles as random factors along the interval 2.5–5 s.

	*%E_k11_*	*%E_k12_*	*%E_k22_*
Parameters	F	p	F	p	F	p
**# stiffness**	>10000	*<0.0001	798.91	*<0.0001	>10000	*<0.0001
**# damping**	1.04	0.41	0.48	0.71	0.89	0.51
**inertia**	23.15	*<0.0001	13.97	*<0.0001	7.43	*<0.0001
**noise**	10.54	*<0.0001	2.66	0.11	>10000	*<0.0001
**condition**	9.18	0.06	10.07	*0.05	11.65	*0.04

* Statistically significant parameters.

### Estimation of eigenvectors

**Figure 10:**
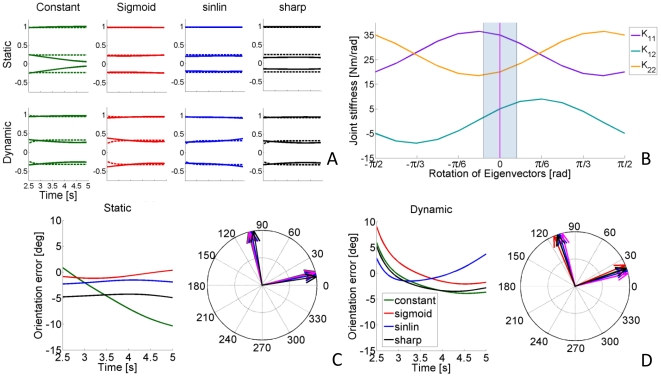
Representation of the eigenvectors and relative errors during simulations. A) *FIRST ROW:* Representation of the time-invariant coefficients of the eigenvector matrix 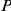 (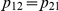) for the static simulations. Dashed lines represent the coefficient of the imposed matrix 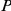 and solid lines represent the estimated 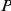 for the different stiffness time profiles: constant (green), sigmoidal (red), sinlin (blue), sharp (black). *SECOND ROW:* The coefficients of matrix 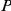 and their estimations for the dynamic case, where the variation of hand position makes the coefficients time-varying. B) Effect of misestimating the orientation of 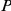 on the stiffness coefficients. The estimations presented in this work are within the shaded blue area. C) *RIGHT:* Representation of the eigenvectors at the beginning of the estimation (time = 2.5 s) for the postural case. Coefficients of the imposed matrix 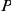 are in magenta. *LEFT:* Error in the eigenvector orientation throughout the estimation time window. The reference eigenvectors are shown in magenta. D) Same as panel B for the dynamic condition.

In general, the matrix of the eigenvectors 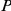 of the system in (31) changes over time during movements and is constant during posture (Figure 9). In the specific case simulated and presented here, the eigenvectors of the stiffness matrix 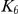 do not change in either the dynamic or the static cases, because each coefficient of the stiffness matrix 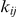 is multiplied by the same weight function 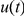 and varies proportionally to all the others (53). However, 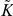 depends on the inertial matrix 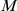 (26) which depends on arm configuration; therefore, in the dynamic case, each eigenvector pair of 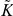 varies along the trajectory as a consequence of the changing arm configuration, The variation in the orientation of the eigenvectors along the movements however is small, and comparable in magnitude to the intrinsic estimation errors of the proposed technique. The variation of the eigenvector orientation along the trajectory is presented in Figure 10b,d which shows that in both the static and dynamic cases the maximum error on the eigenvector orientation is below 10°. Indeed, mis-estimating the orientation of 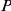 is equivalent to rotating the stiffness matrix through an angle equal to the error. It is useful to recall that a rotation of the stiffness matrix does not change the intrinsic properties of the elastic field associated with it. The ellipses associated to the stiffness matrix will retain the same shape but will simply be rotated, which will have a direct influence on the estimation of the stiffness parameters. The percentage error induced in the stiffness matrix coefficients estimations depends on the initial orientation of 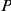. The maximum error on the diagonal terms is found for a rotation of 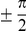 with respect to the initial orientation, where 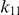 becomes 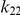 and vice-versa (Figure 10c). The terms outside of the diagonal can be strongly influenced and in general present larger percentage errors due to the non-linear transformations of these coefficients, and to their small magnitude. However, mis-estimating the stiffness coefficients outside the diagonal will only reflect the orientation of the stiffness ellipses without changing the intrinsic properties of the associated elastic field.

**Figure 11:**
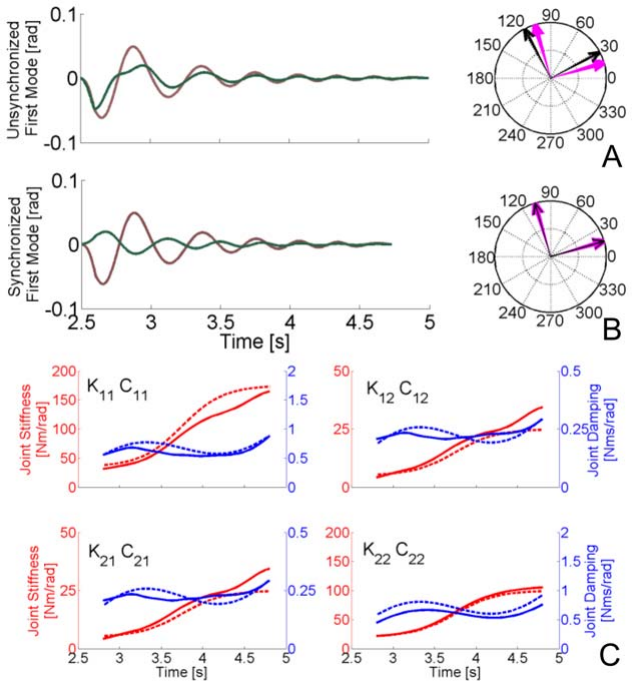
Mode Synchronization and parameter estimation with complex modes. A) LEFT: unsynchronized first mode. RIGHT: imposed (Magenta) vs. estimated (Black) eigenvectors using unsynchronized modes. The error is equal to half the rotation imposed on the damping matrix to simulate non-classical damping. B) Synchronized modes and eigenvector respectively. C) Estimation of stiffness and damping for a sigmoid-sinlin Stiffness-Damping time-profile, when signals are corrupted with signal dependent noise.

When the stiffness and damping matrices 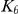 and 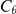 are rotated by the angle 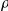 with respect to each other, the matrix 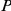 does not represent the eigenvectors for both 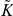 and 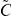. The different modes oscillating at the same angular frequency 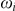 are out of phase by the angle 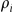. If we were to estimate 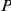 by applying (35) without synchronizing the two modes, we would obtain a matrix 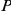 rotated by either 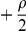 or 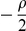 depending on which matrix (i.e. 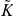 or 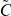) had been taken as a reference (Figure 11a). By synchronizing the two modes we ensure that 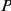 is representative of both 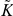 and 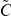 (Figure 11b), and can be used to reconstruct 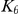 and 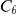 using (48), (26) and (18). An example of such a reconstruction is shown in Figure 11c. To simulate the behavior of our proposed reconstruction in a particularly unfavorable condition, we implemented a KV system with the stiffness matrix changing sigmoidally, and the damping matrix initially rotated by 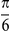 with respect to the stiffness matrix changing in a “sinlin” temporal profile. This reproduces experimental observations that stiffness and damping can present peaks at different instants along the movement trajectory [23]. The signals were corrupted with signal dependent noise as described above in the methods section. The percentage RMS error on the complete estimated stiffness and damping profiles, shown in Figure 11 for this particular condition were 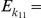
14.1%, 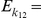
17.7%, 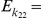
4.5%, 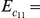
12.0%, 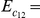
11.0%, and 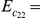
16.0%.

### Normalized force in Non-Linear and Higher order Systems

**Figure 12:**
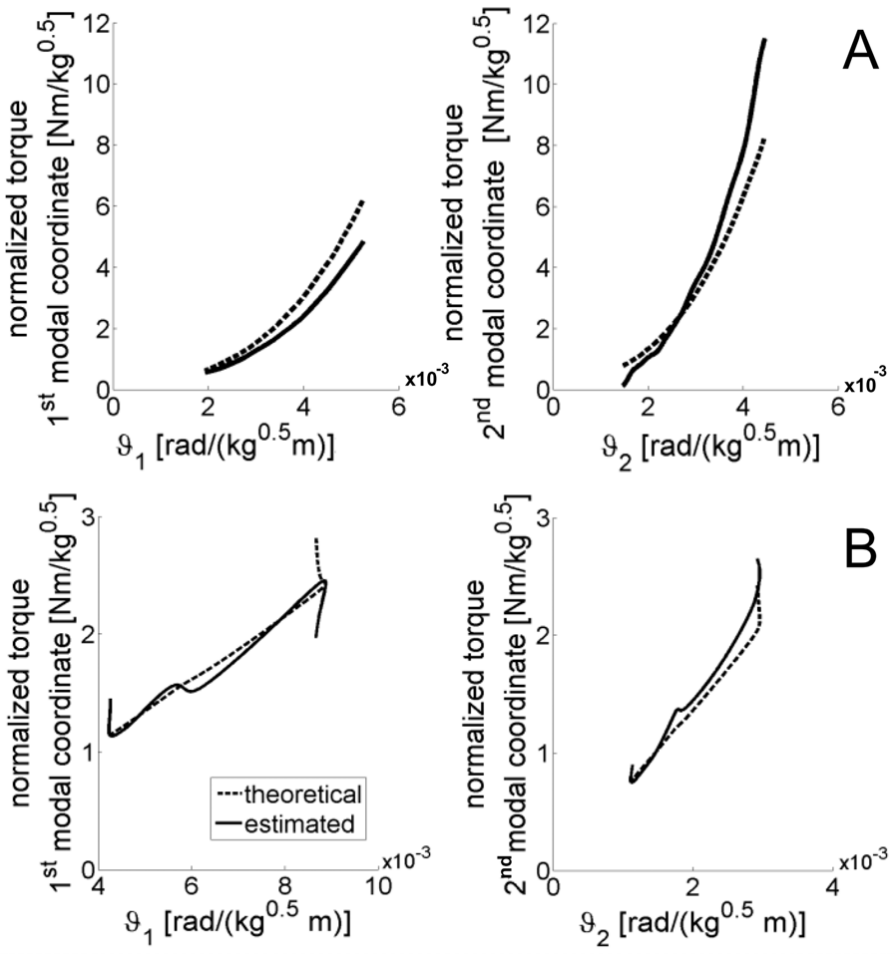
Estimation of normalized force for non-linear and higher-order systems. A) Estimation of normalized force for the Duffing model for 2 DOF. Solid line represents estimation, while dashed line depicts the imposed value. B) Estimation of normalized force for the Poynting-Thomson model.

We investigated how well our technique identified the nature of non-linear (Duffing) and higher than second-order (Poynting-Thomson) systems. The simulations for both cases were carried out in a dynamic condition, using a perturbation directed along the y axis. The inertial parameters were computed from anthropometric data reported in Table 1, using the estimation method proposed by Zatsiorsky [62]. A constant noise (SNR = 10 dB) was also added. Extracting the lumped coefficients of the models would require a numerical optimization, but we could calculate the viscoelastic force of the system by means of the instantaneous time-frequency variables 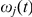 and 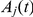 as specified in (49). Errors in the estimation of the lumped parameters would be affected by the estimation of the viscoelastic force and the inherent approximation introduced by the numerical optimization. We used the former as a quantifier of the fit of our method. Figure 12 compares the theoretical normalized viscoelastic force with the time-frequency estimates, to which correspond the following RMS percentage errors: Duffing: 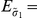
23.1%, 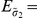
19.4%; Poynting-Thomson: 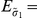
18.4%, 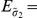
26.9%.

## Discussion

We have presented a new technique for estimating arm viscoelastic characteristics during both static postural and movement conditions. Estimations are based on spectral decomposition and modal testing principles and use a brief (5 N-20 ms) force pulse to estimate the mechanical behavior of the upper limb during free response. The technique does not require assumptions of stationarity, ergodicity, or linearity. The estimation of the viscoelastic components, stiffness and damping, do not require movements and tasks to be repeated over time but can be carried out for a single test trial.

For linear second order systems, simulations of postural and forward reaching tasks were analyzed, imposing non-linearly time-varying stiffness and damping profiles. The estimation of stiffness and damping parameters was achieved using modal analysis, thus solving an inverse vibrational problem instant by instant where both the eigenvalues and the eigenvectors of the vibrational system were measured. Eigenvalues (i.e. the natural frequencies of the mechanical system) were evaluated by analyzing a reassigned spectrogram in the time-frequency domain, specifically identifying the instantaneous vibrational frequencies as a function of time. The eigenvectors (i.e. the vibrational modes) of the system were evaluated using an approach based on the separability of the modes' time series (the frequency of each mode is sufficiently different from the others to be separately identifiable). The separation of each mode was obtained by filtering the free response signals around the resonant frequencies of the system. The adequacy of our method was evaluated using a noise sensitivity analysis, also including signal dependent noise which is common in biological systems. Non-linear and higher order systems were also analyzed by means of the aforementioned time-frequency spectral decomposition during forward reaching movements, corrupted by high level noise. The characteristics of the system intrinsic viscoelastic fields were identified non-parametrically. Modeling nonlinear dynamical systems can be quite challenging and the results can be affected by error growth. Observational errors in measurements of the underlying system can also be amplified by the system dynamics [83].

Many different approaches have been employed for estimating stiffness and damping of moving limbs, spanning from a simple regression between kinetic and kinematic variables following a set of force perturbations to the sophisticated use of displacement servo-perturbations and auto-regressive models. In general, the use of a servo-displacement perturbation as opposed to a force perturbation makes it easier to obtain unbiased estimates of system parameters when performing the linear regression between the perturbation and the elicited force at the hand. Regressive techniques reported in the literature employed perturbations that lasted for at least 200 ms. Several perturbations in different directions are necessary to obtain one regression estimate on a single trajectory point, thus repetition of the task is a requirement. Moreover, when the stiffness estimation is performed at multiple points along a trajectory, numerous blocks of movement repetitions are necessary. These features represent a serious limitation of regressive techniques, especially when the viscoelastic components need to be estimated during continuously changing non-repeatable processes.

A fundamental difference between regressive techniques and our time-frequency approach resides in the number of perturbations required to obtain a complete estimation of stiffness and damping. Even for a simple stationary system, the reliance of regressive techniques on multiple perturbations is essential because the force (or displacement) generated by the displacement (or force) perturbation is being measured. This theoretically requires a minimum of 4 perturbed trials (3 if the system is symmetric) to estimate stiffness and damping at a single point in the whole trajectory. Furthermore, several trials are required to estimate the unperturbed movement trajectory that is used as a reference for the application of the perturbations. By contrast, our modal analysis is able to extract from a single impulsive response the information necessary to estimate stiffness and damping along a whole trajectory (as opposed to a single point) by analyzing the frequency and decay of the oscillation. This capability holds true also for a non-stationary system where frequency and amplitude changes can be tracked as a function of time. The length of the estimation time window is also an important factor. Using a vibrational approach, in non-conservative systems, the energy injected by the perturbation will be dissipated within a specific amount of time. The response signal components with higher frequency tend to have lower amplitude, and thus lower power content, and are more rapidly attenuated by the damping.

The sensitivity of the time-frequency technique can diminish when the estimation of the viscoelastic characteristics is performed long after the onset of the perturbation. To predict accurately the system characteristics, the energy injected into the system cannot be completely dissipated within the analysis time window. For longer estimation windows, perturbations with higher energy should be used, but not so high that subjects can become aware of the perturbations and voluntarily modify the system characteristics, thereby compromising the identification process. The analysis presented here demonstrates that considering ranges of stiffness and damping reported in the literature, our technique can accurately estimate the viscoelastic characteristics of a time varying coupled linear system well beyond 2 s after the onset of the perturbation.

Tracking stiffness and damping changes using regressive techniques requires multiple perturbations at different instants. This is particularly critical when trying to identify a system with fast dynamics and sudden changes in stiffness and damping. The Nyquist–Shannon sampling theorem specifies that to identify a change in a viscoelastic variable occurring at a frequency 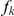, the time gap between the points at which the variable is to be identified must be smaller than the Nyquist period 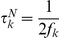. By contrast, our technique does not require multiple perturbations to identify fast changing dynamics of the viscoelastic field and is able to track fast dynamic changes such as the “sharp” presented here. Estimation accuracy does tend to degrade as the changes in stiffness and damping become faster because of the filtering effect of the spectrogram, when several sliding windows are averaged together.

Arm stiffness can be influenced by three separate factors: the intrinsic stiffness of muscles and tendons, the level of voluntary co-contraction, and the intervention of reflexes. Our technique's capacity for continuous stiffness estimation following a perturbation duration of only 20 ms separates these three components because they tend to predominate in different epochs of a movement. For example, we can observe the influences of each factor as the estimated stiffness changes in time: the intrinsic stiffness is mostly dependent upon the biomechanics of the limb which will influence the stiffness estimation right after the perturbation is applied; stretch sensitive reflexes, usually act on a specific time scale between 70 and 150 ms after the perturbation onset, and their effects are visible on the stiffness estimation with a 50 ms delay [14], [84]. This temporal segmentation implies that whatever is estimated more than 200 ms after the perturbation onset may be influenced by voluntary control. Hence, by analyzing how stiffness evolves in time, the effect of each control loop can be studied in single trials. The rapidity of this estimation is thus suitable for identifying stiffness time-profiles during movement adaptation paradigms, thereby providing a fundamental tool to identify motor control strategies. The capacity to monitor variations in stiffness and damping during single trials may particularly benefit the study of rehabilitation training. During robotic therapy, an assistive force field is applied to the limbs of impaired individuals to supply the minimal amount of force necessary to assist them in completing motor tasks. The force is then diminished on a trial-by-trial basis to help the subject regain independence. During such a procedure, the modulation of the assistive force field is different from trial to trial (i.e. non-repeatable motor tasks) and a low fatigue threshold may limit the number of trials the subject can perform [85], [86], [87], [88]. The robotic manipulandum can be a viable tool to estimate limb stiffness, but its utility has so far been restricted by the limitations of regressive techniques. Our proposed approach avoids these limitations.

The technique we propose also has applications in the study of motor adaptation to novel environments where constant, velocity-, and acceleration-dependent force fields are present [89], [90], [91]. The object of analysis often is to capture stiffness and other characteristics in individual perturbed movements, such as the initial perturbed movement or single catch trials. Our approach also applies to cases where a subject adapts to an inertial force field delivered without mechanical contact at the arm [90], because the perturbations needed for modal time-frequency analysis are introduced without robotic devices. The modal analysis techniques can be applied on a trial-by-trial basis to monitor how stiffness varies during motor adaptation.

## Supporting Information

Supplement S1
**Stiffness and Damping Asymmetry due to Dynamic effects.** The asymmetry of the dynamic matrix 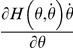, which is a component of the joint stiffness 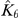, is analyzed.(PDF)

Supplement S2 **Tables of estimation methods statistical comparison.** Tables showing the Stiffness and Damping RMS percentage errors of all estimation methods for KV models during posture and movements. Errors are computed both along the entire stiffness profile and between the first two estimation points used with the regressive methods. The influence of different inertial models for the case sigmoid-sinlin-SDN (K-C-noise), is also shown.
(PDF)

Supplement S3 **Third order form of the PT model and conditions for oscillatory free response.** Analysis of the third-order PT model's oscillatory behavior: a limited region of non-oscillatory free response exists for a ratio between the stiffness of the tendon and the stiffness of the muscle fibers greater than eight.
(PDF)

Supplement S4 **Dynamics zero of the PT model impulse response.** An analytical demonstration on the presence of a dynamic zero for the third order PT model.
(PDF)
