# Supplementary material for: Measuring Multi-Joint Stiffness during Single Movements: Numerical Validation of a Novel Time-Frequency Approach
Source: PLoS One. 2012 Mar 20;7(3):e33086. doi: 10.1371/journal.pone.0033086 (PMC3309009; doi:10.1371/journal.pone.0033086)
Supplement: Supplement S1 — Stiffness and Damping Asymmetry due to Dynamic effects. The asymmetry of the dynamic matrix , which is a component of the joint stiffness , is analyzed. (PDF) [file pone.0033086.s001.pdf]

Supplement S1. Stiffness and Damping Asymmetry due to Dynamic effects

Recalling equation 3 that the differential equation representing the rigid body kinetic of the double pendulum model in Figure 1 is:

$$M(\theta)\ddot{\theta} + H(\theta,\dot{\theta})\dot{\theta} + G(\theta) = \tau_{in}(D^n\theta) + \tau_{ext}(t) \tag{S1}$$

where the inertial matrix is

$$M(\theta,t) = \begin{bmatrix} \kappa + 2\beta c_2 & \chi + \beta c_2 \\ \chi + \beta c_2 & \chi \end{bmatrix} \tag{S2}$$

and the Coriolis matrix is

$$H(\theta,\dot{\theta}) = \begin{bmatrix} -\beta s_2\dot{\theta}_2 & -\beta s_2(\dot{\theta}_1 + \dot{\theta}_2) \\ \beta s_2\dot{\theta}_1 & 0 \end{bmatrix} \tag{S3}$$

where the body segment parameters κ, β and χ are constants, and $s_2 = \sin(\theta_2)$ and $c_2 = \cos(\theta_2)$.

The internal force field $\vec{\psi}(\delta(D^1\theta),t)$ depends on the derivatives with respect to the kinematics of the Coriolis and inertial matrix, namely:

$$\vec{\psi}(\delta(D^1\theta),t) = \left(\frac{\partial H(\theta,\dot{\theta})}{\partial\dot{\theta}} + H(\theta,\dot{\theta})\right)\delta\dot{\theta} + \left(\frac{\partial M(\theta)\ddot{\theta}}{\partial\theta} + \frac{\partial H(\theta,\dot{\theta})\dot{\theta}}{\partial\theta}\right)\delta\theta \tag{S4}$$

Developing the matrix derivatives we obtain:

$$\begin{aligned} \frac{\partial H(\theta,\dot{\theta})}{\partial\theta_1} &= [0] \\ \frac{\partial H(\theta,\dot{\theta})}{\partial\theta_2} &= \begin{bmatrix} -\beta c_2\dot{\theta}_2 & -\beta c_2(\dot{\theta}_1 + \dot{\theta}_2) \\ \beta c_2\dot{\theta}_1 & 0 \end{bmatrix} \end{aligned} \tag{S5}$$

$$\frac{\partial H(\theta,\dot{\theta})}{\partial \dot{\theta}_1} = \begin{bmatrix} 0 & -\beta s_2 \\ \beta s_2 & 0 \end{bmatrix}$$
$$\frac{\partial H(\theta,\dot{\theta})}{\partial \dot{\theta}_2} = \begin{bmatrix} -\beta s_2 & -\beta s_2 \\ 0 & 0 \end{bmatrix} \tag{S6}$$

$$\frac{\partial M(\theta)}{\partial \theta_1} = [0]$$
$$\frac{\partial M(\theta)}{\partial \theta_2} = \begin{bmatrix} -2\beta s_2 & -\beta s_2 \\ -\beta s_2 & 0 \end{bmatrix} \tag{S7}$$

Substituting S5-S7 in S4 allows us to see the asymmetry of $\vec{\psi}\left(\delta\left(D^1\theta\right),t\right)$ matrix coefficients:

$$\begin{aligned}
&\frac{\partial H(\theta,\dot{\theta})\dot{\theta}}{\partial \dot{\theta}} + H(\theta,\dot{\theta}) = \\
&= \left[\begin{bmatrix} 0 & -\beta s_2 \\ \beta s_2 & 0 \end{bmatrix}\begin{Bmatrix} \dot{\theta}_1 \\ \dot{\theta}_2 \end{Bmatrix} \quad \begin{bmatrix} -\beta s_2 & -\beta s_2 \\ 0 & 0 \end{bmatrix}\begin{Bmatrix} \dot{\theta}_1 \\ \dot{\theta}_2 \end{Bmatrix}\right] + \begin{bmatrix} -\beta s_2\dot{\theta}_2 & -\beta s_2\left(\dot{\theta}_1 + \dot{\theta}_2\right) \\ \beta s_2\dot{\theta}_1 & 0 \end{bmatrix} = \\
&= \begin{bmatrix} -\beta s_2\dot{\theta}_2 & -\beta s_2\dot{\theta}_1 - \beta s_2\dot{\theta}_2 \\ \beta s_2\dot{\theta}_1 & 0 \end{bmatrix} + \begin{bmatrix} -\beta s_2\dot{\theta}_2 & -\beta s_2\left(\dot{\theta}_1 + \dot{\theta}_2\right) \\ \beta s_2\dot{\theta}_1 & 0 \end{bmatrix} = \\
&= \begin{bmatrix} -2\beta s_2\dot{\theta}_2 & -2\beta s_2\left(\dot{\theta}_1 + \dot{\theta}_2\right) \\ 2\beta s_2\dot{\theta}_1 & 0 \end{bmatrix}
\end{aligned} \tag{S8}$$

$$\begin{aligned}
&\frac{\partial M(\theta)\ddot{\theta}}{\partial \theta} + \frac{\partial H(\theta,\dot{\theta})\dot{\theta}}{\partial \theta} = \\
&= \left[\begin{bmatrix} 0 & 0 \\ 0 & 0 \end{bmatrix}\begin{Bmatrix} \ddot{\theta}_1 \\ \ddot{\theta}_2 \end{Bmatrix} \quad \begin{bmatrix} -2\beta s_2 & -\beta s_2 \\ -\beta s_2 & 0 \end{bmatrix}\begin{Bmatrix} \ddot{\theta}_1 \\ \ddot{\theta}_2 \end{Bmatrix}\right] + \\
&+ \left[\begin{bmatrix} 0 & 0 \\ 0 & 0 \end{bmatrix}\begin{Bmatrix} \dot{\theta}_1 \\ \dot{\theta}_2 \end{Bmatrix} \quad \begin{bmatrix} -\beta c_2\dot{\theta}_2 & -\beta c_2\left(\dot{\theta}_1 + \dot{\theta}_2\right) \\ \beta c_2\dot{\theta}_1 & 0 \end{bmatrix}\begin{Bmatrix} \dot{\theta}_1 \\ \dot{\theta}_2 \end{Bmatrix}\right] = \\
&= \left[\begin{Bmatrix} 0 \\ 0 \end{Bmatrix} \quad \begin{Bmatrix} -2\beta s_2\ddot{\theta}_1 - \beta s_2\ddot{\theta}_2 \\ -\beta s_2\ddot{\theta}_1 \end{Bmatrix}\right] + \left[\begin{Bmatrix} 0 \\ 0 \end{Bmatrix} \quad \begin{Bmatrix} -\beta c_2\dot{\theta}_2\dot{\theta}_1 - \beta c_2\left(\dot{\theta}_1 + \dot{\theta}_2\right)\dot{\theta}_2 \\ \beta c_2\dot{\theta}_1^2 \end{Bmatrix}\right] = \\
&= \begin{bmatrix} 0 & -2\beta s_2\ddot{\theta}_1 - \beta s_2\ddot{\theta} - \beta c_2\dot{\theta}_2\dot{\theta}_1 - \beta c_2\left(\dot{\theta}_1 + \dot{\theta}_2\right)\dot{\theta}_2 \\ 0 & -\beta s_2\ddot{\theta}_1 + \beta c_2\dot{\theta}_1^2 \end{bmatrix}
\end{aligned} \tag{S9}$$
