# Supplementary material for: Measuring Multi-Joint Stiffness during Single Movements: Numerical Validation of a Novel Time-Frequency Approach
Source: PLoS One. 2012 Mar 20;7(3):e33086. doi: 10.1371/journal.pone.0033086 (PMC3309009; doi:10.1371/journal.pone.0033086)
Supplement: Supplement S2 — Tables of estimation methods statistical comparison. Tables showing the Stiffness and Damping RMS percentage errors of all estimation methods for KV models during posture and movements. Errors are computed both along the entire stiffness profile and between the first two estimation points used with the regressive methods. The influence of different inertial models for the case sigmoid-sinlin-SDN (K-C-noise), is also shown. (PDF) [file pone.0033086.s002.pdf]

Supplement S2. Tables of estimation methods statistical comparison

Table S1 shows that the stiffness estimation error using our method is comparable to the regression methods. However, one of the most important features of our spectrogram technique is that while still maintaining a comparable accuracy to regression methods it is capable of estimating time varying stiffness profiles with only one perturbation, compared to the tens of repeatable perturbed movements necessitated by regressive techniques.

Estimation accuracy tends to degrade for all four methods as the changes in stiffness and damping become faster. For the regressive methods, this depends on the number of estimates among the time-profile. As the frequency of each parameter variation increases, a larger number of points are necessary to track its change, as a consequence of the Nyquist-Shannon theorem. For the spectrogram method, the accuracy of the estimation in time is a trade off with the accuracy in the frequency domain. Increasing the resolution in the frequency domain limits the number of points available in the time domain. The use of a Reassigned Spectrogram (RS) mitigates this limitation. For the fastest stiffness profile change ("sharp" profile) the rising time of stiffness or damping is $t_r = 0.3s$, and the spectrogram method outperformed the other methods for the estimation of k_{22} and c_{22}.

In the first half of the dynamic case estimations, both slower (sigmoid) and faster (sinlin) variation of stiffness are well tracked. The sensitivity of the spectrogram technique to measures in the simulated "sinlin" condition diminishes towards the end of the movement, and as a result the increase of stiffness (and therefore of natural frequency) toward the end of the movement can be difficult to observe. This result is consistent with the observation that when a set amount of energy is injected into a mechanical system, the response signal components with higher frequency tend to have lower amplitude, and thus lower power content, and are more rapidly attenuated by the damping. To obviate this problem a set of perturbations with higher energy content can be used.

Tables S2 and S4 reveal that the time-frequency method outperformed regressive methods when estimating damping time-profiles. Since the damping is extracted independently of the stiffness

(equation (44) does not depend upon the resonant frequency), small damping values can be clearly identified. On the other hand, full regression techniques need to separate the contributions of damping and stiffness and are more prone to rounding errors when the dependent variables are quite different in magnitude, as in this case when the viscous force is small compared to the elastic force.

Table S5 illustrates the effect that the inertia has on estimations of stiffness and damping using the spectrogram technique and the regressive methods. A significant case is presented here where stiffness and damping vary asynchronously and the noise is signal-dependent. To properly compare the results of our spectrogram technique with the regressive methods, we limited our identification to one estimate per condition, obtained with a perturbation applied in the direction of positive Y (see Figure 1). Although full-regression methods can estimate the inertia of a mechanical system, the influence of a change in the inertial parameters on the estimation of stiffness and damping is substantial. The inertial models HV and DE produce inertial parameters that cause all the estimation methods we compared to perform poorly.

Table S1: Stiffness RMS percentage errors for KV models during posture and movements for entire stiffness profile.

		STATIC						DYNAMIC					
		$\% E_{K_{11}}$		$\% E_{K_{12}}$		$\% E_{K_{22}}$		$\% E_{K_{11}}$		$\% E_{K_{12}}$		$\% E_{K_{22}}$	
Method	***K Profile*** ↓	Min	Max	Min	Max	Min	Max	Min	Max	Min	Max	Min	Max
Spectrogram	const	4.97	12.4	7.25	49.7	0.85	21.2	5.0	20.9	3.19	49.2	3.03	18.2
	sigmoid	8.18	14.5	6	25.2	0.87	23.4	5.8	18	7.11	40.4	1.06	17.1
	sinlin	8.34	13.1	3.58	14.6	0.64	19.2	12.8	22.9	4.53	29.7	1.3	16.8
	sharp	15.6	21.5	22	44.3	7.68	10.8	13.5	20.6	26.2	76.5	7.67	15.5
Displ. Full Reg.	const	0.03	0.09	0.64	1.68	0.2	19.6	0.94	1.1	8.17	9.78	1.01	19.2
	sigmoid	5.53	5.62	4.6	5.22	2.82	16	5.2	5.29	7.12	7.61	6.72	15.6
	sinlin	2.83	2.85	2.85	3.04	2.59	19.4	3.04	3.11	7.17	8.17	3.15	18.9
	sharp	29	29.3	29.6	30.1	17.1	30.7	29.8	29.9	30	31.3	30.3	30.8
Force Full Reg.	const	4.28	6.59	2.04	8.58	0.15	20.4	3.36	6.41	13.5	19.8	4.19	25.8
	sigmoid	3.85	5.58	3.63	5.7	2.73	21.6	2.53	3.27	26.8	30.5	3.54	23.9
	sinlin	3.52	4.09	3.11	3.5	2.55	20.6	3	3.63	12.7	16.1	3.69	24.1
	sharp	16.8	16.9	17.2	19.1	17.1	30.5	20.3	21.2	54.5	63.5	16.9	18.5
Disp. Steady St..	const	0.0	0.0	0.3	1.1	1.0	19.2	0.7	1.0	1.0	4.1	1.0	19.1
	sigmoid	5.6	5.6	4.8	5.4	6.4	15.8	6.0	6.3	6.1	8.1	6.3	15.8
	sinlin	2.2	2.2	2.3	2.4	2.4	19.3	2.3	2.4	2.4	3.5	2.5	19.3
	sharp	62.3	62.8	61.7	62.4	63.6	63.9	63.4	63.7	63.4	66.8	63.4	63.8

Minimum and maximum percentage errors are presented as a function of stiffness profiles and estimation methods.

Table S2 Damping RMS percentage errors for KV models during posture and movements for entire stiffness profile.

		STATIC						DYNAMIC					
		% $E_{C_{11}}$		% $E_{C_{12}}$		% $E_{C_{22}}$		% $E_{C_{11}}$		% $E_{C_{12}}$		% $E_{C_{22}}$	
Method	***K Profile*** ↓	Min	Max	Min	Max	Min	Max	Min	Max	Min	Max	Min	Max
Spectrogram	const	2.0	12.0	10.3	37.4	1.8	11.9	5.9	27.8	9.6	93.2	3.1	25.4
	sigmoid	9.5	24.3	5.1	48.3	1.4	14.2	8.4	32.3	12.8	148.1	3.4	34.4
	sinlin	3.8	17.2	17.8	49.8	6.7	12.0	5.4	20.2	19.4	58.7	6.0	16.9
	sharp	14.9	41.2	18.6	74.0	6.6	22.4	18.4	60.7	41.3	128.6	7.4	21.6
Displ. Full Reg.	const	0.2	18.6	4.1	35.2	0.7	18.5	16.8	106.1	39.5	431.2	17.1	124.3
	sigmoid	56.1	146.8	10.2	105.4	46.8	155.3	61.1	303.1	48.6	1190.4	67.2	371.3
	sinlin	40.2	78.5	29.0	60.0	35.3	74.6	38.4	194.4	38.5	1113.0	67.4	337.3
	sharp	89.5	235.3	18.6	156.8	68.2	200.8	84.9	330.4	54.2	1123.3	87.0	380.1
Force Full Reg.	const	32.0	70.2	17.1	90.0	4.6	17.9	5.8	26.2	88.6	192.2	16.3	27.7
	sigmoid	29.1	57.6	16.4	65.6	23.7	49.2	42.7	110.1	105.0	336.2	24.9	54.9
	sinlin	21.2	35.4	19.7	43.6	21.5	35.3	26.8	56.0	68.5	197.5	23.3	39.9
	sharp	54.6	127.3	19.1	119.5	44.4	114.8	84.3	205.7	187.8	436.4	40.0	76.5

Minimum and maximum percentage errors are presented as a function of stiffness profiles and estimation methods.

Table S3 Stiffness RMS percentage errors for KV models during posture and movements between the first two estimation points of the regressive methods.

		STATIC						DYNAMIC					
		$\% E_{K_{11}}$		$\% E_{K_{12}}$		$\% E_{K_{22}}$		$\% E_{K_{11}}$		$\% E_{K_{12}}$		$\% E_{K_{22}}$	
Method	***K Profile*** ↓	Min	Max	Min	Max	Min	Max	Min	Max	Min	Max	Min	Max
Spectrogram	const	2.0	10.9	9.8	48.2	0.8	19.6	6.0	19.8	4.2	69.5	1.0	14.6
	sigmoid	3.1	10.5	5.8	39.8	1.0	23.7	2.4	13.0	3.2	62.2	1.5	17.1
	sinlin	10.8	15.1	2.2	18.8	0.2	20.7	13.7	17.5	1.8	42.0	0.4	16.8
	sharp	4.0	8.5	4.7	44.3	0.5	6.0	3.0	16.3	4.0	85.0	0.7	15.7
Displ. Full Reg.	const	0.0	0.1	0.9	1.7	0.5	19.6	2.0	2.2	17.9	20.7	1.6	18.4
	sigmoid	5.2	5.3	3.8	4.4	5.9	15.3	3.0	3.2	14.1	15.9	3.7	15.7
	sinlin	2.2	2.3	1.2	1.8	2.8	17.8	0.9	1.0	10.8	13.0	1.1	16.2
	sharp	0.3	0.3	0.9	1.6	0.7	1.0	2.1	2.4	20.1	22.4	1.7	11.7
Force Full Reg.	const	4.8	6.6	2.2	8.3	0.3	20.4	6.2	8.7	5.0	9.7	0.6	22.9
	sigmoid	3.0	4.7	1.8	5.4	1.9	18.3	2.0	2.6	19.6	25.2	1.2	21.5
	sinlin	4.3	4.9	2.6	3.5	1.9	21.3	2.0	2.5	12.9	17.8	2.5	22.5
	sharp	5.1	6.6	2.2	8.3	0.4	0.5	7.2	9.2	7.3	12.5	0.6	4.0
Disp. Steady St..	const	0.0	0.0	0.3	1.1	1.0	19.2	0.3	1.0	1.1	5.7	0.1	19.3
	sigmoid	5.6	5.7	4.7	5.4	6.7	14.7	5.4	6.5	3.3	12.5	6.2	14.8
	sinlin	1.3	1.3	0.4	0.9	2.1	18.3	1.5	2.0	1.2	4.0	1.8	18.4
	sharp	0.0	0.0	0.3	1.1	1.0	1.2	0.1	0.9	0.9	7.8	0.7	2.0

Minimum and maximum percentage errors are presented as a function of stiffness profiles and estimation methods.

Table S4 Damping RMS percentage errors for KV models during posture and movements between the first two estimation points of the regressive methods.

		STATIC						DYNAMIC					
		% $E_{C_{11}}$		% $E_{C_{12}}$		% $E_{C_{22}}$		% $E_{C_{11}}$		% $E_{C_{12}}$		% $E_{C_{22}}$	
Method	***K Profile*** ↓	Min	Max	Min	Max	Min	Max	Min	Max	Min	Max	Min	Max
Spectrogram	const	0.7	12.7	5.1	55.5	2.6	13.9	9.7	27.2	1.8	50.6	1.5	23.9
	sigmoid	3.8	16.2	2.6	57.7	0.2	13.0	0.4	28.9	11.8	95.9	4.5	34.2
	sinlin	2.7	17.7	1.0	82.1	6.5	19.8	0.2	13.4	6.1	86.4	6.4	26.5
	sharp	2.2	11.4	5.7	83.4	1.5	8.5	4.5	32.8	9.0	102.6	1.5	22.1
Displ. Full Reg.	const	0.2	10.7	1.8	13.8	0.2	11.2	29.7	159.0	25.9	517.3	30.8	186.4
	sigmoid	31.9	49.9	21.1	33.0	26.0	41.9	45.8	203.9	49.2	608.9	59.9	263.3
	sinlin	60.6	124.0	45.3	116.7	52.9	125.8	59.7	310.2	37.3	1436.0	127.0	547.7
	sharp	0.1	9.8	2.7	14.3	0.1	10.1	28.0	127.5	26.7	519.0	27.5	164.7
Force Full Reg.	const	33.5	70.2	18.6	91.5	3.8	7.1	2.6	24.3	84.7	291.1	17.8	23.8
	sigmoid	38.6	75.1	21.5	86.1	20.9	35.5	27.9	47.8	24.0	127.2	34.4	57.8
	sinlin	35.0	61.3	31.9	73.3	33.7	60.9	30.5	71.3	43.1	156.0	37.0	72.1
	sharp	33.5	70.2	18.4	91.4	3.9	8.2	2.6	26.0	86.4	307.1	15.9	23.2

Minimum and maximum percentage errors are presented as a function of stiffness profiles and estimation methods.

Table S5: Influence of different inertial models for the case sigmoid-sinlin-SDN (K-C-noise).

Methods	Inertial model ↓	% $E_{K_{11}}$	% $E_{K_{12}}$	% $E_{K_{22}}$	% $E_{C_{11}}$	% $E_{C_{12}}$	% $E_{C_{22}}$
Spectrogram	HV	13.9	50.2	9.0	26.9	73.5	28.9
	DE	29.7	76.8	13.5	21.5	120.8	46.8
	CH	5.0	15.5	4.1	11.0	35.2	20.4
	CL	4.8	15.6	5.3	41.2	51.3	33.3
	MC	5.8	23.7	7.6	21.4	64.0	17.1
	Z2	5.8	16.5	3.2	10.6	26.7	21.9
	PI	12.7	29.6	7.1	12.4	43.1	21.9
	Z1	5.3	17.6	3.7	13.4	36.4	25.4
	DL	8.6	17.2	5.5	7.4	60.2	18.8
Disp. Full Regress.	HV	8.1	24.3	16.2	98.0	407.7	169.3
	DE	7.9	23.8	15.9	139.5	806.4	224.9
	CH	4.7	14.5	6.3	113.2	482.8	143.8
	CL	4.9	13.3	6.7	65.7	174.0	86.7
	MC	5.1	15.0	8.8	119.3	548.3	156.7
	Z2	4.7	14.4	6.1	111.2	471.5	142.2
	PI	4.7	14.2	6.2	141.4	699.7	186.9
	Z1	4.7	14.8	6.4	102.4	408.1	129.6
	DL	4.8	14.5	7.4	122.1	570.1	159.0
Force Full Regress.	HV	6.1	115.9	39.9	45.8	87.5	58.5
	DE	5.4	108.3	39.4	39.4	64.2	49.0
	CH	4.9	23.9	7.4	37.4	116.4	23.6
	CL	5.6	27.3	7.9	39.5	107.7	32.6
	MC	4.8	39.3	13.1	38.4	98.8	30.0
	Z2	4.8	23.0	7.1	36.9	113.1	23.7
	PI	4.6	21.8	7.0	33.2	111.0	21.2
	Z1	5.1	24.9	7.6	38.1	118.1	25.0
	DL	4.7	29.0	9.5	35.9	104.5	25.4
Displ. Steady State	HV	9.1	26.6	10.1	N/A	N/A	N/A
	DE	8.9	25.4	10.0	N/A	N/A	N/A
	CH	5.2	6.4	5.7	N/A	N/A	N/A
	CL	5.3	6.6	5.8	N/A	N/A	N/A
	MC	5.8	8.3	6.9	N/A	N/A	N/A
	Z2	5.2	6.4	5.8	N/A	N/A	N/A
	PI	5.2	6.0	5.9	N/A	N/A	N/A
	Z1	5.4	9.6	5.5	N/A	N/A	N/A
	DL	5.3	7.8	6.4	N/A	N/A	N/A

The table reports stiffness and damping RMS percentage errors. The following abbreviations are used for the inertial methods: Hanavan (HV), Dempster (DE), Chandler (CH), Clauser (CL), McConville (MC), Zatsiorsky and Seluyanov (1983) (Z1), Water Immersion (PI), Zatsiorsky and Seluyanov (2002) (Z2), de Leva (DL).
