# Supplementary material for: Measuring Multi-Joint Stiffness during Single Movements: Numerical Validation of a Novel Time-Frequency Approach
Source: PLoS One. 2012 Mar 20;7(3):e33086. doi: 10.1371/journal.pone.0033086 (PMC3309009; doi:10.1371/journal.pone.0033086)
Supplement: Supplement S3 — Third order form of the PT model and conditions for oscillatory free response. Analysis of the third-order PT model's oscillatory behavior: a limited region of non-oscillatory free response exists for a ratio between the stiffness of the tendon and the stiffness of the muscle fibers greater than eight. (PDF) [file pone.0033086.s003.pdf]

Supplement S3.Third order form of the PT model and conditions for oscillatory free response

Recalling equation (14) from the main text

$$M\delta\ddot{\theta}+\psi+f(t)=g(t)$$

During a free response, the external force is null, hence $g(t)=0$. Furthermore, without loss of theoretical rigor, the internal force field ψ generated by the mechanism's dynamics can be assumed negligible. Therefore equation (14) can be rewritten as follows:

$$M\delta\ddot{\theta}+f(t)=0 \qquad \text{(S10)}$$

where the viscoelastic force of the PT system, expressed in equation (16) in the main text, is the only internal force

$$f(t)=-\frac{K_\theta^S\cdot C_\theta^P}{K_\theta^S+K_\theta^P}\cdot\delta\dot{\theta}-\frac{K_\theta^S\cdot K_\theta^P}{K_\theta^S+K_\theta^P}\cdot\delta\theta-\frac{C_\theta^P}{K_\theta^S+K_\theta^P}\cdot\dot{f}(t)$$

By simple arithmetic and substitution, we can isolate the time derivative $\dot{f}(t)$ of the internal viscoelastic force field $f(t)$.

$$\dot{f}(t)=\frac{M}{C_\theta^P}\left(K_\theta^S+K_\theta^P\right)\delta\ddot{\theta}+K_\theta^S\cdot\delta\dot{\theta}+\frac{K_\theta^S\cdot K_\theta^P}{C_\theta^P}\cdot\delta\theta \qquad \text{(S11)}$$

Furthermore, we can derive both members of (S10) to obtain:

$$M\delta\dddot{\theta}+\dot{f}(t)=0 \qquad \text{(S12)}$$

Substituting (S11) in (S12) we obtain the third-order formulation of the PT model:

$$M\delta\dddot{\theta} + \frac{M}{C_\theta^P}\left(K_\theta^S + K_\theta^P\right)\delta\ddot{\theta} + K_\theta^S\delta\dot{\theta} + \frac{K_\theta^S \cdot K_\theta^P}{C_\theta^P}\delta\theta = 0 \qquad \text{(S13)}$$

The following substitutions can be applied to (S13):

$$\begin{aligned} A &= \left(K_\theta^S + K_\theta^P\right) \\ B &= K_\theta^S \\ C &= K_\theta^S \cdot K_\theta^P \end{aligned} \qquad \text{(S14)}$$

We can represent the characteristic polynomial of the differential equation (S13) in the following form:

$$M\lambda^3 + \frac{M}{C_\theta^P}A\lambda^2 + B\lambda + \frac{C}{C_\theta^P} = 0 \qquad \text{(S15)}$$

The solution of the characteristic polynomial can be obtained using Cardano's method . Two auxiliary variables can be defined:

$$\begin{aligned} p &= -\left(\frac{A}{3C_\theta^P}\right)^2 + \frac{B}{3M} \\ q &= \left(\frac{A}{3C_\theta^P}\right)^3 - \frac{AB}{6MC_\theta^P} + \frac{C}{2MC_\theta^P} \end{aligned} \qquad \text{(S16)}$$

The discriminant $\Delta = q^2 + p^3$ of the cubic equation can be computed. The sign of the discriminant determines the type of solution for equation (S15). The roots of a cubic polynomial can be:

- Three distinct real roots ($\Delta < 0$)
- Three real roots, at least two of which are equal ($\Delta = 0$)
- One real root and two complex conjugate roots ($\Delta > 0$)

We will demonstrate that the sign of the discriminant depends on the ratio between the tendon stiffness K_θ^S and the muscle stiffness K_θ^P. A PT model presents an oscillating free response only if the solution of (S15) includes a complex root, i.e. if $\Delta > 0$.(see fig.S2)

From (S16), the discriminant can be expressed as a function of the muscle damping as follows:

$$\Delta\left(C_\theta^P\right) = q^2 + p^3 = \frac{1}{108\left(C_\theta^P\right)^4 M^3}\left(4B\left(C_\theta^P\right)^4 - M\left(A^2B^2 + 18ABC - 27C^2\right)\left(C_\theta^P\right)^2 + 4M^2A^3C\right) \qquad \text{(S17)}$$

By solving (S17) for $\Delta\left(C_\theta^P\right) > 0$ we will find the region in the solution domain where the model presents an oscillating free response. The multiplicative term outside the main parenthesis in (S17) is always positive, and the expression within parenthesis is a bi-quadratic form. Hence, to find the solutions to $\Delta = 0$, we can substitute $\left(C_\theta^P\right)^2 = v$ and solve the following quadratic equation:

$$Q(v) = \left(4Bv^2 - M\left(A^2B^2 + 18ABC - 27C^2\right)v + 4M^2A^3C\right) = 0 \qquad \text{(S18)}$$

To solve (S18) we can impose the following two conditions :

$$\begin{cases} \Delta_Q = M^2(AB - 9C)^3(AB - C) \geq 0 \\ \left(A^2B^2 - 18ABC - 27C^2\right) \geq 0 \end{cases} \qquad \text{(S19)}$$

The expression (S19a) represents the condition of the discriminant of (S18) to be non-negative, since $v = \left(C_\theta^P\right)^2 \geq 0$. The equation (S19b) can be clarified by putting (S18) in its monic form:

$$v^2 - (v_1 + v_2)v + (v_1 \cdot v_2)v = 0 \qquad \text{(S20)}$$

The coefficient of the first degree term needs to have the same sign as the sum of the two solutions (which are both positive since $C_\theta^P \geq 0$).

Since $M^2 > 0$ and $AB - C = \left(K_\theta^S + K_\theta^P\right)K_\theta^S - K_\theta^S \cdot K_\theta^P = \left(K_\theta^S\right)^2 > 0$, the condition expressed in (S19a) is equivalent to the following:

$$\left(AB - 9C\right) \geq 0 \text{, i.e. } \left(K_\theta^S + K_\theta^P\right)K_\theta^S - 9 \cdot K_\theta^S \cdot K_\theta^P \geq 0 \qquad \text{(S21)}$$

Assuming the stiffness of the tendon proportional to the stiffness of the muscle, we can define $K_\theta^S = n \cdot K_\theta^P$, with $n > 0$. Substituting in (S21) we obtain

$$n(n-8)\left(K_\theta^P\right)^2 \geq 0 \Leftrightarrow n \geq 8 \qquad \text{(S22)}$$

This shows that for $n < 8$ equation (S15) has two complex conjugate roots, which translate in a free oscillatory response of the PT system, independently of the value of C_θ^P. On the other hand, if $n \geq 8$ there exists a finite interval of damping values within which the system does not present an oscillatory free response. Figure S1 depicts a numerical example where K_θ^P=70 Nm/rad, M=0.2 Kg/m^2 and $n = 5$, 8, and, 11.

The parameters in the implementation of the PT model used in our simulations are compatible with those presented by Kistemaker and Rozendaal [46], including $n = 5$. The value of C_θ^P used in our implementation (35 Nms/rad) would guarantee an oscillatory free response of the PT model for $n \geq 8$ as well, although such a high value of n justifies the approximation of the mechanical system with a second order Kelvin-Voigt model.

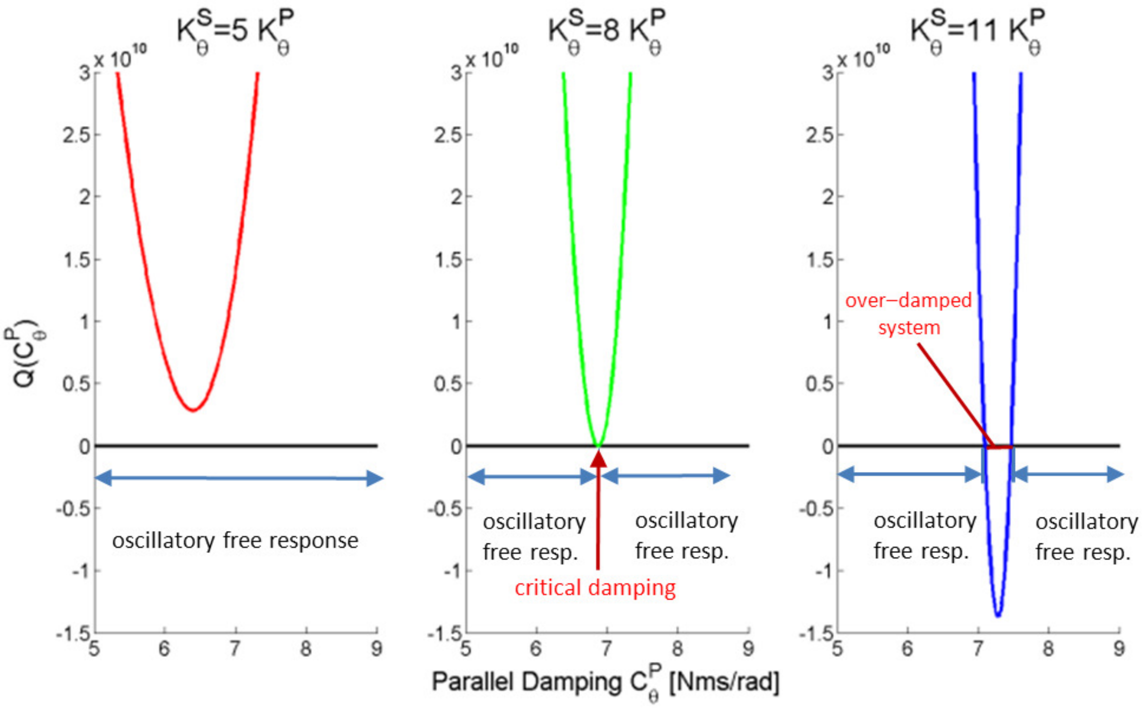

Figure S1

Figure S1: Sign of the characteristic polynomial's discriminant for a Poynting-Thomson model. The discriminant of the characteristic polynomial of a Poynting-Thomson model is shown as a function of the muscle damping C_θ^P and the parameter n, which represents the proportionality constant between the stiffness of the tendon and muscle elements. The function is shown for K_θ^P =70 Nm/rad, M =0.2 Kg/m^2 and $n = 5$, 8, and, 11. For $n < 8$ the discriminant is positive, independently of the value of C_θ^P, which translate in a free oscillatory response of the PT system. If $n \geq 8$ there exists a finite interval of damping values within which the system does not present an oscillatory free response.
