# Supplementary material for: Measuring Multi-Joint Stiffness during Single Movements: Numerical Validation of a Novel Time-Frequency Approach
Source: PLoS One. 2012 Mar 20;7(3):e33086. doi: 10.1371/journal.pone.0033086 (PMC3309009; doi:10.1371/journal.pone.0033086)
Supplement: Supplement S4 — Dynamics zero of the PT model impulse response. An analytical demonstration on the presence of a dynamic zero for the third order PT model. (PDF) [file pone.0033086.s004.pdf]

Supplement S4. Dynamics zero of the PT model impulse response.

Dividing both members of equation (4) by the Laplace transform of the displacement function we can calculate the transfer function of the impulse response, which is the solution of equation (S13)

$$\mathrm{M}s^2 + \frac{\Sigma(s)}{\mathrm{X}(s)} = \frac{\mathrm{G}(s)}{\mathrm{X}(s)} \qquad \text{(S23)}$$

With reference to the PT model shown in Figure (2), we can define the transfer function of the muscle fibers as $Z_\theta^P = C_\theta^P s + K_\theta^P$. The transfer function of the whole model is the transfer function of the series of tendon and muscle fibers:

$$\frac{\Sigma(s)}{\mathrm{X}(s)} = \frac{K_\theta^S Z_\theta^P}{\left(K_\theta^S + Z_\theta^P\right)} \qquad \text{(S24)}$$

Substituting and rearranging (S24) in (S23) we obtain:

$$\frac{\mathrm{G}(s)}{\mathrm{X}(s)} = \frac{K_\theta^S\left(C_\theta^P s + K_\theta^P\right)}{C_\theta^P s + \left(K_\theta^P + K_\theta^S\right)} + \mathrm{M}s^2 \qquad \text{(S25)}$$

Since the transfer function of a unit impulse response is $\mathrm{G}(s) = 1$, the Laplace transform of the impulse response (solution of S13) is as follows

$$X(s) = \frac{1}{\mathrm{M}} \frac{s + \frac{\left(K_\theta^S + K_\theta^P\right)}{C_\theta^P}}{s^3 + \frac{\left(K_\theta^S + K_\theta^P\right)}{C_\theta^P} s^2 + \frac{K_\theta^S}{\mathrm{M}} s + \frac{\left(K_\theta^S \cdot K_\theta^P\right)}{C_\theta^P \mathrm{M}}} \qquad \text{(S26)}$$

$$X(s) = \frac{1}{\mathrm{M}} \frac{s + z}{s^3 + bs^2 + cs + d}$$

and presents a dynamic zero.
